# m^6^A Methylation Modification Patterns and Tumor Microenvironment Infiltration Characterization in Pancreatic Cancer

**DOI:** 10.3389/fimmu.2021.739768

**Published:** 2021-09-20

**Authors:** Mengyu Sun, Meng Xie, Tongyue Zhang, Yijun Wang, Wenjie Huang, Limin Xia

**Affiliations:** ^1^Department of Gastroenterology, Institute of Liver and Gastrointestinal Diseases, Tongji Hospital of Tongji Medical College, Huazhong University of Science and Technology, Wuhan, China; ^2^Hubei Key Laboratory of Hepato-Pancreato Biliary Diseases, Tongji Hospital, Tongji Medical College, Huazhong University of Science and Technology, Wuhan, China; ^3^Hepatic Surgery Center, Tongji Hospital, Tongji Medical College, Huazhong University of Science and Technology, Wuhan, China

**Keywords:** pancreatic cancer, m^6^A, tumor microenvironment, immunotherapy, mutation burden

## Abstract

Recent studies have shown that RNA N^6^-methyladenosine (m^6^A) modification plays an important part in tumorigenesis and immune-related biological processes. However, the comprehensive landscape of immune cell infiltration characteristics in the tumor microenvironment (TME) mediated by m^6^A methylation modification in pancreatic cancer has not yet been elucidated. Based on consensus clustering algorithm, we identified two m^6^A modification subtypes and then determined two m^6^A-related gene subtypes among 434 pancreatic cancer samples. The TME characteristics of the identified gene subtypes were highly consistent with the immune-hot phenotype and the immune-cold phenotype respectively. According to the m^6^A score extracted from the m^6^A-related signature genes, patients can be divided into high and low m^6^A score groups. The low score group displayed a better prognosis and relatively strong immune infiltration. Further analysis showed that low m^6^A score correlated with lower tumor mutation burden and PD-L1 expression, and indicated a better response to immunotherapy. In general, m^6^A methylation modification is closely related to the diversity and complexity of immune infiltration in TME. Evaluating the m^6^A modification pattern and immune infiltration characteristics of individual tumors can help deepen our understanding of the tumor microenvironment landscape and promote a more effective clinical practice of immunotherapy.

## Introduction

More than 160 RNA modifications including N^7^-methylguanine (m^7^G), N^6^-methyladenosine (m^6^A), and N^5^-methylcytosine (m^5^C) have been identified. These modifications play a significant role in regulating RNA fate (1). In eukaryotes, m^6^A is regarded as the most important and abundant mRNA modification, accounting for more than 80% of all RNA methylation modifications (2). It is now clear that m^6^A methylation exists in almost all types of RNA, including coding RNA and non-coding RNA (3). The m^6^A modification is catalyzed by RNA methyltransferases such as METTL3, METTL14, METTL16, WTAP, VIRMA, ZC3H13, RBM15, and RBM15B (writers), while the modification is removed by demethylases such as FTO and ALKBH5 (erasers). In addition, modifications can be recognized by m^6^A binding proteins, such as YTHDC1/2, YTHDF1/2/3, HNRNPC, FMR1, LRPPRC, HNRNPA2B1, IGFBP1/2/3, and RBMX (readers) (4–6). A growing body of evidence shows that m^6^A regulators are involved in vital biological processes and are dynamically regulated in many physiological and pathological processes (7–9). Abnormal expression and genetic alterations of m^6^A regulators are closely related to events such as developmental defects, metabolic disorders, abnormal immune regulation, and tumor progression (10, 11). A comprehensive understanding of potential m^6^A regulators’ expression perturbation and genetic variation under cancer heterogeneity will facilitate the identification of therapeutic targets based on RNA methylation (12, 13).

Pancreatic cancer (PC) is a highly lethal malignant tumor, which is also one of the leading causes of cancer death worldwide (14). In the past 25 years, its global burden has more than doubled (15). With the in-depth comprehending of pathology, the diversity and complexity of tumor microenvironment have been increasingly understood, and immune cell subgroups which intimately involved in tumor genesis, metastasis and treatment are gradually recognized (16–19). Tumor microenvironment (TME) has been found to play an important role in ineffective treatment and poor prognosis of pancreatic cancer. Many molecules and related signal transduction pathways in the microenvironment can promote cancer metastasis or immunosuppression. A variety of soluble immunosuppressive molecules and immunosuppressive cells can lead to the disorder of immune effector cells, thus forming a unique immunosuppressive environment for pancreatic cancer (20). Increasing research focuses on whether the components of TME (including acellular matrix, pancreatic stellate cells, immune cells and soluble factors) can be used as effective targets for PC therapy (21). Recently, immune checkpoint inhibitors and chimeric antigen receptor T (CAR-T) cell therapy have become popular immunotherapies related to TME in pancreatic cancer (22, 23). However, whether these therapies can bring clinical benefits remains to be fully studied. A comprehensive understanding of the characteristics of the tumor microenvironment in PC will make a beneficial contribution to the research of immunotherapy and provide new insights for basic and clinical applications.

An increasing number of studies have confirmed the close correlation between TME infiltrating immune cells and m^6^A modification, which could not be completely explained by the mechanism of RNA degradation. According to the study of Liu et al. (24), FTO enhances protein expression by regulating the m^6^A modification of JUNB and CEBPB genes, thereby promoting tumor glycolysis and inhibiting T cell effects. The FTO inhibitor Dac51 can inhibit FTO-mediated demethylation, inhibit the glycolytic ability of tumor cells, increase T cell infiltration, and have a synergistic effect with anti-PD-L1 therapy. The study of Han et al. (25) showed that YTHDF1 recognizes and binds to the transcript encoding lysosomal protein modified by m^6^A, increases the translation of lysosomal cathepsin in dendritic cells (DC), while inhibition of cathepsin can significantly increase the ability of cross-presenting antigen of dendritic cells. The absence of YTHDF1 in DC can enhance the cross-presentation of tumor antigens and the cross-priming of CD8+ T cells, thereby increasing the anti-tumor response of CD8+ T cells and enhancing the therapeutic effect of PD-L1 checkpoint blockade. It has been reported that cytotoxic tumor-infiltrating CD8+ T cell is increased in METTL3 or METTL14 deficient tumor. Depletion of METTL3 and METTL14 can inhibit m^6^A modification and enhance the response to anti-PD-1 therapy in colorectal cancer and melanoma (26). However, the above studies have focused on one or two m^6^A regulators and immune cell types, while tumor formation and suppression are the results of the highly synergistic effects of multiple regulatory factors. Therefore, the comprehensive analysis of the infiltration characteristics of tumor microenvironment mediated by m^6^A regulator is helpful to promote the cognition of tumor immune regulation.

At present, there are widely accepted molecular subtypes in pancreatic cancer, such as two tumor-specific subtypes and stromal subtypes identified by Moffitt et al. and purity Independent Subtyping of Tumors (PurIST) developed by Rashid et al. (27, 28). These classifications may mainly be concerned with the components of the tumor at the pathological level and show good clinical value. We aimed to identify new subtypes from the m^6^A methylation modification direction and construct scores to supplement the existing clinical variable information, and correlate these analyses with the tumor microenvironment. Meng et al. have proposed an m^6^A-related mRNA signature, which can play a good prognostic predictive effect in pancreatic cancer (29). However, their study divided groups based on the existence of alterations (mutation and/or CNV) of m^6^A-related genes, and then identified differentially expressed genes for model construction. According to previous studies (30), data including gene expression profile, somatic mutation, and DNA methylation information can be used to identify the primary sites and origins of tumors, but the accuracy of gene expression data is the highest, especially in pancreatic cancer. Since gene expression data may provide more clinical value, this study identified subtypes based on m^6^A regulator gene expression to mine more accurate clinical subtypes and prognostic indicators.

In this study, we integrated the transcriptome information of 434 pancreatic cancer samples from five independent cohorts, comprehensively evaluated m^6^A modification patterns, and correlated the characteristics of immune cell infiltration in TME. Through the unsupervised clustering method, we identified two different m^6^A modification subtypes and defined two m^6^A-related gene subtypes. We found that distinct subgroups were accompanied with different immune cell infiltration characteristics. In addition, we constructed a scoring scheme to quantify individual m^6^A modification patterns, and predicted the prognosis and response to immunosuppressive therapy based on the score. Our findings indicate that m^6^A modification is closely related to TME immune cell infiltration, and can be used as a favorable predictor of prognosis and immunotherapy in pancreatic cancer.

## Materials and Methods

### Collection and Preprocessing of PC Public Datasets

The workflow of this study was shown in **Figure S1**. The expression profile data and clinical information of pancreatic cancer samples were obtained from the Cancer Genome Atlas (TCGA, RRID : SCR_003193, https://tcga-data.nci.nih.gov/tcga/) and Gene-Expression Omnibus (GEO, RRID : SCR_005012, https://www.ncbi.nlm.nih.gov/geo/) database. This study collected 5 independent PC cohorts (TCGA-PAAD, GSE28735, GSE57495, GSE62452, GSE85916) for further analysis. RNA sequencing data in TCGA (FPKM format) were downloaded and transformed into TPMs (transcripts per kilobase million). We download the normalized matrix file in GEO, and applied ComBat algorithm in the R package SVA to eliminate batch effects between different GEO data sets. The survival status and survival time of the samples were extracted from the clinical information of the 5 PC cohorts. Data with follow-up time less than 31 days and duplicate data were excluded. Somatic mutation data was collected from the TCGA database. The copy number variation data (CNV) of TCGA-PAAD was downloaded from the UCSC Xena database (http://xena.ucsc.edu/).

### Unsupervised Clustering of 23 m^6^A Regulators

We searched the relevant literature on m^6^A methylation modification to identify recognized m^6^A regulators for subsequent analysis. A total of 23 m^6^A regulators were included, including 8 writers (METTL3, METTL14, METTL16, WTAP, VIRMA, ZC3H13, RBM15, and RBM15B), 13 readers (YTHDC1, YTHDC2, YTHDF1, YTHDF2, YTHDF3, HNRNPC, FMR1, LRPPRC, HNRNPA2B1, IGFBP1, IGFBP2, IGFBP3, and RBMX), and 2 erasers (FTO and ALKBH5). Unsupervised clustering analysis was performed to identify different m^6^A methylation modification subtypes according to the expression of 23 m^6^A regulators, and patients were classified for further analysis. The number of clusters (K) and their stability were determined by the consensus clustering algorithm. Li et al. (31) tested four methods for finding K: the cumulative distribution function (CDF), the proportional change in the area under the CDF curve upon an increase of K (Δ(K)), GAP-PC (32) and CLEST (33). They found that CDF was able to reveal the correct K, as the CDF curve was flat only for the true K, reflecting a perfectly or near-perfectly stable partitioning of the samples at the correct K. So we took the K corresponding to the flattest CDF curve as the determined number of clusters. The R package consusclusterplus was utilized to perform the above steps (34).

### Estimation of Immune Infiltrating Cells in TME

The R package CIBERSORT was used to quantify the infiltration of different immune cells in PC samples from five cohorts. Leukocyte signature matrix (LM22) contains 547 reference genes, which can be used to distinguish 22 human immune cell phenotypes, including various types of T cells, B cells, NK cells, plasma cells, and myeloid subgroups. CIBERSORT is a deconvolution algorithm, which can calculate the proportion of different types of cells in the sample based on LM22 (35). The ESTIMATE algorithm infers the cell density and tumor purity of the tumor based on the transcriptome profile of the sample (36). Tumor tissue with rich immune cell infiltration indicates a higher immune score and lower tumor purity. The R package ESTIMATE was used to evaluate the immune and stromal content (immune and stromal score) in each sample.

### Identification of Differentially Expressed Genes Between Different m^6^A Modified Phenotypes

Previous consensus clustering algorithms divided patients into two different m^6^A modification subtypes based on the expression of 23 m^6^A regulators. R package Limma was used to identify DEGs between the two m^6^A modification clusters with adjusted P value < 0.05.

### Functional and Pathway Enrichment Analyses of DEGs

GO (Gene Ontology) is a crucial bioinformatics tool for annotating and analyzing the biological functions of genes, including MF (molecular function), BP (biological processes), and CC (cellular components). As a database resource, KEGG (Kyoto Encyclopedia of Genes and Genomes) is mainly used to understand the high-level functions and values of biological systems from molecular-level information. To get annotation information and explore the biological functions of the above DEGs, GO and KEGG enrichment analyses were accomplished using clusterProfiler package with a cutoff of p-value < 0.05 and q-value < 0.05.

### Construction of m^6^A Score

To quantify the modification pattern of m^6^A in individual PC patients, we used principal component analysis (PCA) to construct the m^6^A scoring scheme. Firstly, univariate Cox regression model was performed on DEGs identified between different m^6^A modification clusters, and the genes with significant prognosis effect were selected for clustering samples and constructing m^6^A score. The patients were divided into several groups for further analysis. The number and stability of gene clusters were determined by consensus clustering algorithm. Subsequently, principal component analysis was used to construct the m^6^A-related gene signature, and principal component 1 and principal component 2 were extracted as signature scores. This method focuses the score on the set with the largest block of strongly related or anti-related genes, while reducing the contribution of genes that are not tracked with other set members. Besides, the PCA algorithm can effectively achieve data dimensionality reduction and largely retain the information of the original data. We used a method similar to GGI (37, 38) to define the m^6^A score: $m6Ascore=\sum (PC1_{i}+PC2_{i})$ where i is the expression of final genes related to the m^6^A phenotype.

### Verification of the m^6^A Score

To verify the reliability and clinical application value of m^6^A score, the receiver operating characteristic (ROC) curves of 1-, 3-, and 5-year were drawn. We first drew the ROC curve based on all samples. Then, the ROC curve was drawn solely in the TCGA-PAAD cohort, and the prognostic prediction performance of m^6^A score and other clinical indicators were compared. Univariate and multivariate Cox regression analyses were used to evaluate the correlation between the patient’s m^6^A score, clinical variables, and prognosis to determine whether the score can be used as an independent prognostic indicator of pancreatic cancer. P < 0.05 indicated that the difference was statistically significant. The results were shown in the forest diagram. Next, 8 indicators (age, gender, grade, stage, stage_T, stage_N, stage_M, and m^6^A score) were used to construct a nomogram to personally predict the 1-year, 3-year, and 5-year survival rates of patients. The ROC curve was drawn to show the predictive performance of the nomogram. The R packages survival, survminer, timeROC, rms, and regplot are used for calculation and graph drawing.

### Analysis of Genome Mutation Data

We calculated the copy number increase or loss frequency of 23 m^6^A regulators in the TCGA-PAAD cohort, and used the R package Rcircos to draw a copy number variation map of m^6^A regulators on human chromosomes. To determine the tumor mutation burden (TMB), we counted the total number of non-synonymous mutations in the TCGA-PAAD cohort. R package maftools was used to plot the oncoprint of gene mutation.

### Obtain the Prediction Indicators of Immune Response

Immunophenoscore (IPS) is a favorable factor to predict the efficacy of anti-CTLA-4 and anti-PD-1 regimens, which can quantify the determinants of tumor immunogenicity and show the characteristics of tumor immune landscape (39). The score is calculated based on four categories of immune-related genes, including MHC molecules (MHC), immunomodulators (CP), effector cells (EC), and suppressor cells (SC). IPS of samples in TCGA-PAAD were downloaded from the online platform The Cancer Immunome Atlas (TCIA, RRID : SCR_014508, https://tcia.at/home) for further analysis.

### Statistical Analysis

All statistical analysis and graph drawing were completed by R-4.0.3. The Wilcoxon rank-sum test was used for comparison between two groups. Kaplan-Meier survival analysis and univariate Cox regression model were performed to calculate the relationship between m^6^A regulators and prognosis. According to the correlation between m^6^A score and patient survival, R package Survminer was used to repeatedly test all possible cut-off points to obtain the largest rank statistic, and the patients were divided into high and low m^6^A score groups based on the largest selected log-rank statistic. The Kaplan-Meier method was utilized to draw the survival curve for prognostic analysis, and the log-rank test was used to determine the significance of the difference. Spearman correlation analysis and distance correlation analysis were applied for the correlation test. All heat maps were generated by R package pheatmap. All statistical P value were two-tailed, and P < 0.05 was statistically significant.

## Results

### Construction of Genetic Variation, Immune Infiltration, and Prognostic Landscape of m^6^A Regulators

**Figure 1:**
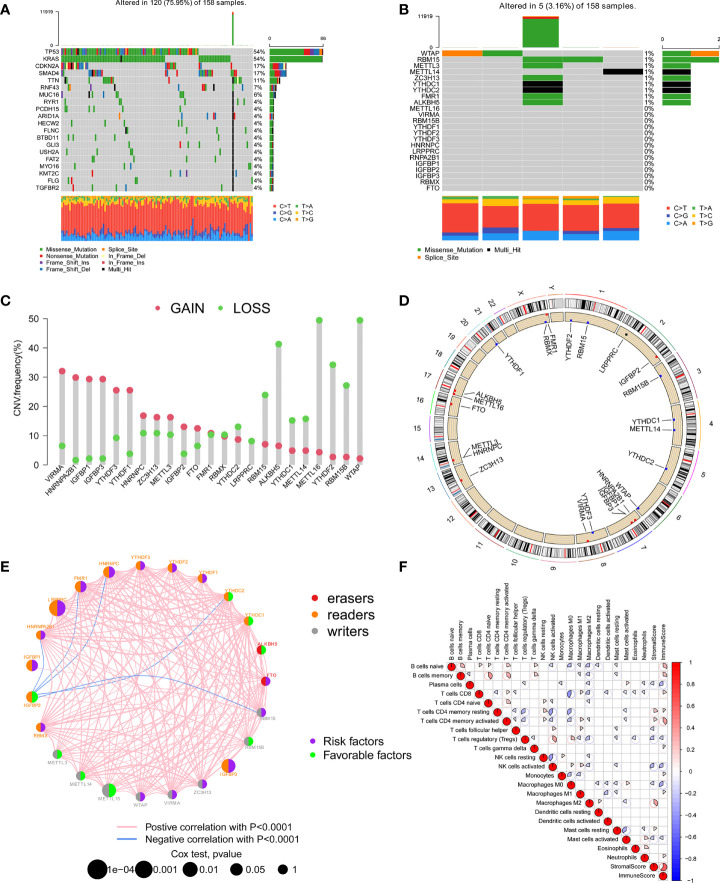
The landscape of genetic variation, immune infiltration, and prognosis of m^6^A regulators. **(A)** The top 20 genes with the highest mutation frequency in the TCGA-PAAD cohort. The main types of mutations were missense mutations. **(B)** Mutations of 23 m^6^A regulators in the TCGA-PAAD cohort. **(C)** The CNV mutation frequency of 23 m^6^A regulators in the TCGA-PAAD cohort. Each column indicated the frequency of mutations. Amplification frequency, red dot; missing frequency, green dot. **(D)** The location of CNV changes of 23 m^6^A regulators on the chromosome. **(E)** The interaction of 23 m^6^A regulators and their prognostic significance in 5 independent PC cohorts. The three types of m^6^A regulatory genes were represented by different colors. Erasers, red; Readers, orange; Writers, grey. The size of the circle represented the prognostic effect of each m^6^A regulator, and was adjusted according to the p-value. Prognostic risk factors, purple; prognostic protective factors, green. **(F)** Cellular interaction of the tumor-infiltrating immune cell types.

In this study, 23 m^6^A regulators were identified, including 8 writers, 13 readers, and 2 erasers. We first calculated the incidence of somatic mutations in PC. Among 158 tumor samples, a total of 120 cases (75.95%) had genetic alterations, of which TP53 and KRAS mutations were the most frequent with more than 50% (**Figure 1A**). However, the mutation frequency of 23 m^6^A regulators was pretty low, genetic alterations occurred in only 5 (3.16%) samples (**Figure 1B**). We further analyzed the relationship between TP53 and KRAS mutations and the expression of m^6^A regulators. There were differences in the expression of multiple m^6^A regulators between the wild group and mutant group (**Supplementary Figures 3, 4**). Analysis of copy number variation of 23 m^6^A regulators showed that CNV mutations were common in PC. VIRMA, HNRNPA2B1, IGFBP1, IGFBP3, YTHDF3, and YTHDF1 had widespread CNV amplification. However, METTL16, WTAP, ALKBH5, YTHDF2, and RBM15B showed extensive CNV deletions (**Figure 1C**). The location of CNV changes on the chromosome was illustrated in **Figure 1D**. Kaplan-Meier survival analysis showed that 15 m^6^A regulators were correlated with the prognosis of PC patients (**Supplementary Figure 2**). Univariate Cox regression model revealed the prognostic value of 23 m^6^A regulators in PC patients (**Supplementary Table 1**). As shown in **Figure 1E**, the network presents a comprehensive landscape of the interactions, connection, and prognostic significance of m^6^A regulators in PC. The results indicated that writers, readers, and erasers have a significant correlation in expression. The cross-talk among them probably plays an essential role in the formation of different m^6^A modification patterns, and might be related to the occurrence and development of cancer. In addition, we implemented CIBERSORT and ESTIMATE algorithms to quantify the activity or enrichment level of immune cells in pancreatic cancer tissues. The correlation coefficient heatmap was used to display a general landscape of immune cell interactions in the tumor microenvironment (**Figure 1F**).

### 23 Regulators-Mediated m^6^A Methylation Modification Subtypes

**Figure 2:**
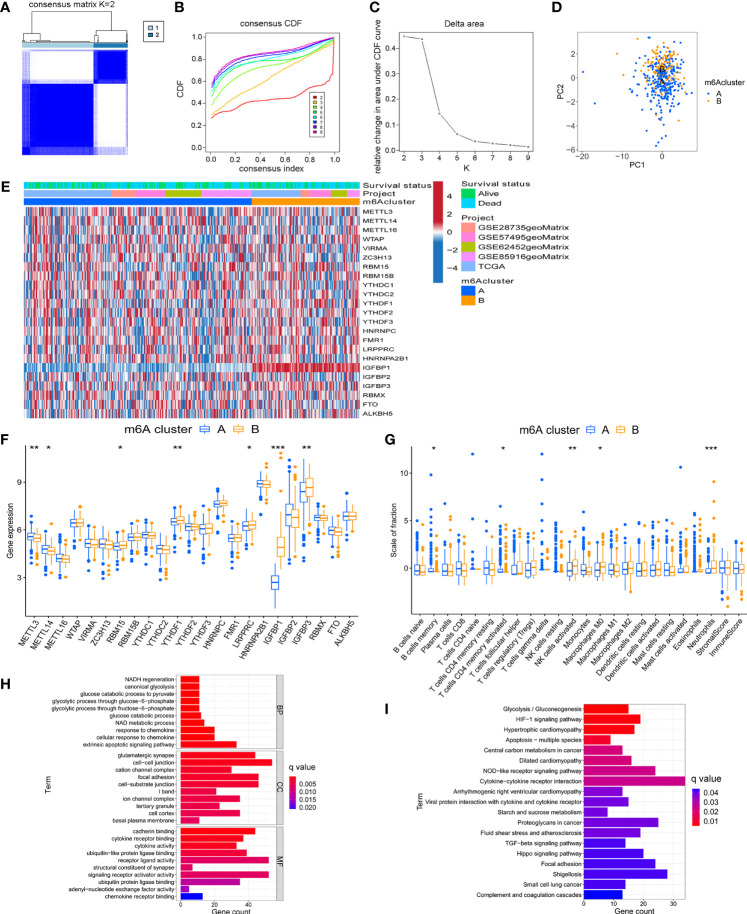
Identification of m^6^A methylation modification subtypes. **(A)** Heat map of sample clustering under k = 2 in 5 independent PC cohorts. **(B)** Consensus clustering cumulative distribution function (CDF) with the number of subtypes k = 2 to 9. **(C)** The relative change of the area under the CDF curve of k = 2 to 9. **(D)** Principal component analysis of the expression profiles of 23 m^6^A regulators to distinguish two determined m^6^A clusters. **(E)** Unsupervised clustering of 23 m^6^A regulators in two m^6^A clusters. **(F)** Differences in the expression of 23 m^6^A regulators between distinct m^6^A clusters. **(G)** TME immune-infiltrating characteristics and transcriptome traits of two m^6^A clusters. **(H)** GO enrichment pathway of differentially expressed genes between two m^6^A clusters. **(I)** KEGG enrichment pathway of differentially expressed genes between two m^6^A clusters. ***P < 0.001; **P < 0.01; *P < 0.05.

Based on the expression of 23 m^6^A regulators, the R package ConensusClusterPlus was used to qualitatively classify patients with different m^6^A modification subtypes. Through the consensus clustering algorithm, two different m^6^A modification subtypes were finally identified, including 294 cases in subtype A and 140 cases in subtype B (**Figures 2A–D** and **Supplementary Table 2**). We named these two subtypes m^6^A cluster A and m^6^A cluster B, showed the expression of 23 m^6^A regulators in the two modified subtypes by heatmap (**Figure 2E**). The expression levels of 23 m^6^A regulators between the two m^6^A clusters were also compared and shown in **Figure 2F**. To explore the internal biological changes under different m^6^A modification modes, we compared the composition of immune cells in TME. The result showed that m^6^A cluster A was characterized by higher infiltration of memory B cells and activated memory CD4+ T cells. In m^6^A cluster B, the infiltration of activated NK cells, M0 macrophages, and Neutrophils were significantly increased (**Figure 2G**). To reveal the potential biomolecular characteristics of different m^6^A modified phenotypes, R package LIMMA was used for differential expression analysis to determine the transcriptome differences between two subtypes. We identified 1159 differentially expressed genes and annotated DEG with R package clusterProfiler. **Figures 2H, I** summarized the significant biological processes of DEG enrichment, such as glucose metabolism, glycolysis, HIF-1 signaling pathway, Hippo signaling pathway, and TGF-β signaling pathway. These results suggested that the m^6^A methylation modification may involve in tumor metabolism and immune regulation, and was closely related to tumor genesis and progression. **Supplementary Tables 3, 4** provides detailed descriptions.

### Identification of m^6^A-Related Gene Subtypes

**Figure 3:**
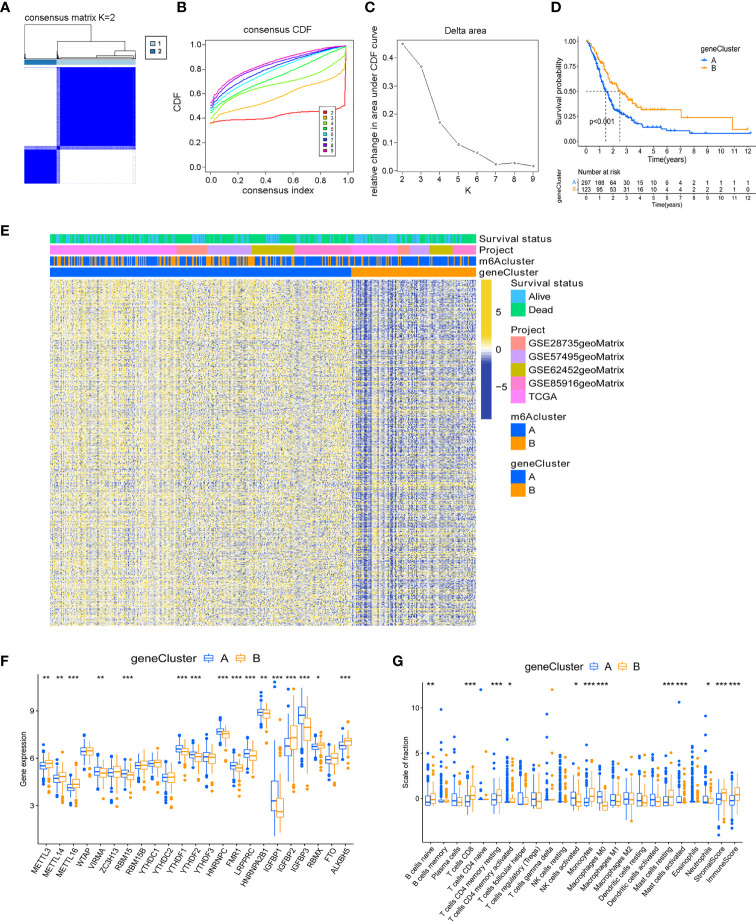
Identification of m^6^A-related gene subtypes. **(A)** Heat map of sample clustering under k = 2 in 5 independent PC cohorts. **(B)** Consensus clustering cumulative distribution function (CDF) with the number of subtypes k = 2 to 9. **(C)** The relative change of the area under the CDF curve of k = 2 to 9. **(D)** Survival analysis of patients in two m^6^A-related gene clusters. **(E)** Unsupervised clustering of m^6^A related signature genes. **(F)** Differences in the expression of 23 m^6^A regulators between distinct gene clusters. **(G)** TME immune-infiltrating characteristics and transcriptome traits of two m^6^A-related gene clusters. ***P < 0.001; **P < 0.01; *P < 0.05.

Although the consensus clustering algorithm based on 23 m^6^A regulators classified PC patients into two subtypes, potential genetic changes and prognostic correlations in these phenotypes were not very clear. We performed univariate COX regression analysis on the 1159 DEGs between the previously identified m^6^A clusters, and obtained 719 survival-related genes which were named m^6^A-related signature genes (**Supplementary Table 5**). Based on representative m^6^A-related signature genes, we adopted unsupervised cluster analysis and identified two stable transcriptome phenotypes, which were defined as gene cluster A and gene cluster B (**Figures 3A–C**). In addition, we explored the prognostic significance of gene subtypes by integrating transcriptome and survival information. Through Kaplan-Meier analysis and log-rank test, gene cluster B showed a better prognosis (P < 0.001, **Figure 3D**). The heatmap showed the transcriptome profile of 719 m^6^A-related signature genes in two gene clusters (**Figure 3E**). The expression levels of 23 m^6^A regulators between the two m^6^A-related gene clusters were also compared. Significant differences in the expression of m^6^A regulators were observed, which was consistent with the expected result of m^6^A modification patterns (**Figure 3F**). Previous studies have shown that the immune system may produce favorable or unfavorable consequences, which may manifest as pro-tumor or anti-tumor activity. Monocytes and anti-tumor lymphocyte subsets such as CD8+ T cells, memory CD4+ T cells, and naive B cells had a higher level of infiltration in gene cluster B, while activated NK cells and mast cells infiltrated more abundantly in gene cluster A (**Figure 3G**). The immune and stromal score based on the ESTIMATE algorithm indicated that the infiltration of immune cells and stromal components in gene cluster B was higher. Therefore, we speculate that the abundant immune cell infiltration in gene cluster B forms an effective anti-tumor immune response.

### Construction of m^6^A Score

**Figure 4:**
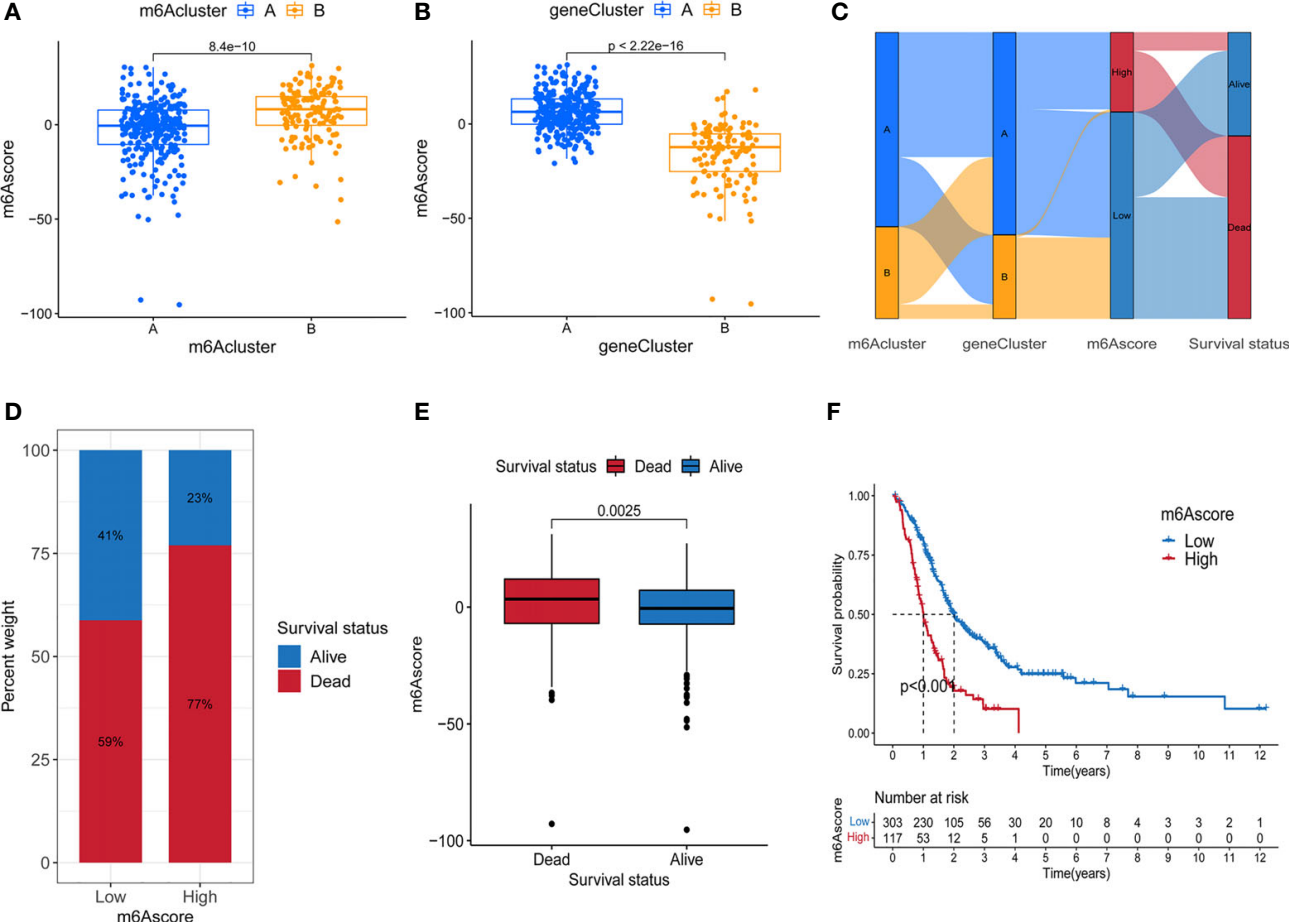
Construction of m^6^A score. **(A)** Difference of m^6^A score between two m^6^A methylation modification subtypes. **(B)** Difference of m^6^A score between two m^6^A-related gene subtypes. **(C)** Alluvial diagram containing m^6^A cluster, gene cluster, m^6^A score and survival changes. **(D)** The proportion of survival and death in high and low m^6^A score group. **(E)** Comparison of m^6^A scores between surviving and dead patients. **(F)** Survival analysis of high and low m^6^A score groups.

Although the above results demonstrated the role of m^6^A methylation modification in the regulation of immune cell infiltration and prognosis, these analyses cannot accurately predict the m^6^A methylation modification pattern in a single tumor patient. To obtain a quantitative index of the m^6^A modification landscape of PC patients, we extracted the scores of principal component 1 and principal component 2 for calculating the final m^6^A score. **Figure 4A** showed that patients’ m^6^A score in m^6^A cluster A was lower than m^6^A cluster B, and **Figure 4B** depicted that the m^6^A score in gene cluster B was lower than that in gene cluster A. We drew an alluvial diagram to display the process of m^6^A score construction (**Figure 4C**). Subsequent analysis revealed the prognostic significance of m^6^A score. According to **Figure 4D**, the patients’ survival rate (41% vs. 23%) in the m^6^A low score group was much higher than that in the high score group. The overall average m^6^A score of the surviving patients was lower than that of the dead patients (**Figure 4E**, P = 0.0025). Kaplan-Meier survival analysis showed that the prognosis of patients in the low m^6^A score group was significantly better (**Figure 4F**, P < 0.001).

### Validation of m^6^A Score and Its Application in Clinical Evaluation

**Figure 5:**
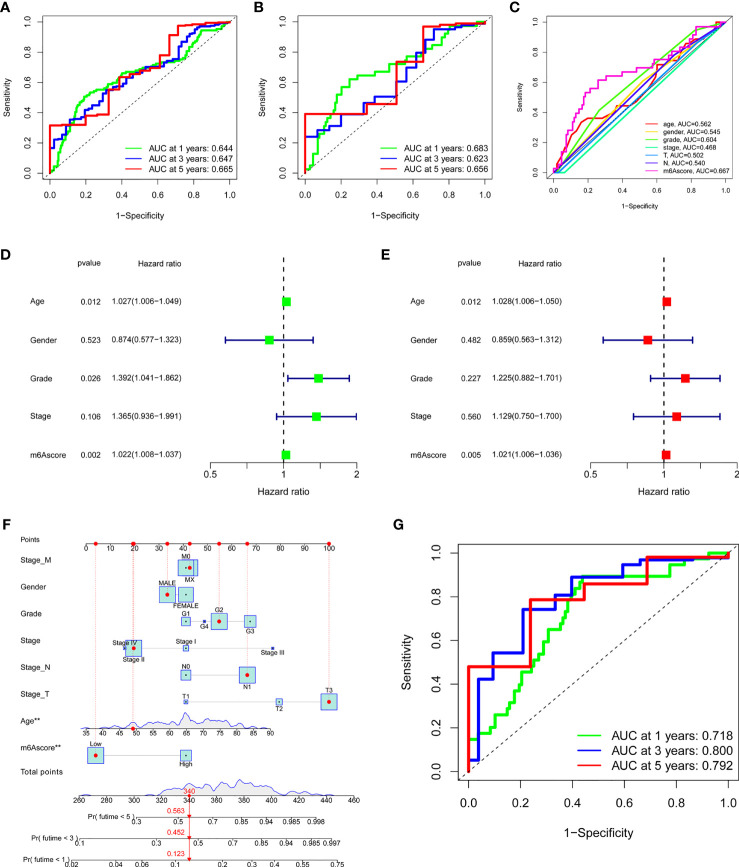
Validation and application of the m^6^A score in the clinical evaluation. **(A)** The AUC values of the 1-year, 3-year, and 5-year ROC curves of m^6^A score in all samples. **(B)** The AUC values of the 1-year, 3-year, and 5-year ROC curves of m^6^A score in the TCGA-PAAD cohort. **(C)** The comparation of 1-year ROC curve with other clinical characteristics in the TCGA-PAAD cohort. **(D)** Univariate COX regression analysis showed that age, grade, and m^6^A score were considered statistically significant. **(E)** Multivariate Cox regression analysis showed age and m^6^A score were independent prognostic predictors. **(F)** The nomogram to predict the probability of 1-year, 3-year, and 5-year survival rate. **(G)** The AUC values of the 1-year, 3-year, and 5-year ROC curves of the nomogram. ***P < 0.001; **P < 0.01; *P < 0.05.

To verify the m^6^A score, the 1-, 3-, and 5-year ROC curves were drawn, and the value of the area under curve (AUC) of the m^6^A score was calculated. The results showed that the AUC values of all three curves were around 0.65 both in total samples (**Figure 5A**) and TCGA-PAAD cohort (**Figure 5B**). We also compared the 1-year ROC curve with other clinical characteristics in TCGA-PAAD cohort, and m^6^A score had the most considerable AUC value (**Figure 5C**). Univariate Cox regression analysis showed that age (p = 0.012, HR = 1.027, 95%CI [1.006-1.049]), grade (p = 0.026, HR = 1.392, 95%CI [1.041-1.862]) and m^6^A score (p = 0.002, HR = 1.022, 95%CI [1.008-1.037]) were considered statistically significant (**Figure 5D**). Multivariate Cox regression analysis showed age (p = 0.012, HR = 1.028, 95%CI [1.006-1.050]) and m^6^A score (p = 0.005, HR = 1.021, 95%CI [1.006-1.036]) were independent prognostic predictors (**Figure 5E**). By integrating multiple clinical indicators, the nomogram can be an effective tool for quantitatively assessing individual risks in the clinical environment. We constructed a nomogram to predict patients’ OS at 1-, 3-, and 5-year. Taking a random sample as an example, the total score of the patient was 340, the probability of survival time less than 1-, 3-, and 5-year were 0.123, 0.452, and 0.563, respectively (**Figure 5F**). The ROC curve was used to evaluate the predictive performance of the nomogram. The AUC values of 1-, 3-, and 5-year ROC curves were 0.718, 0.800, and 0.792, respectively (**Figure 5G**). The results showed that m^6^A score could be used as a new effective clinical predictor and can be combined with other clinical variables to improve the prognosis of patients with pancreatic cancer.

### Correlation Between m^6^A Score and Somatic Variation

**Figure 6:**
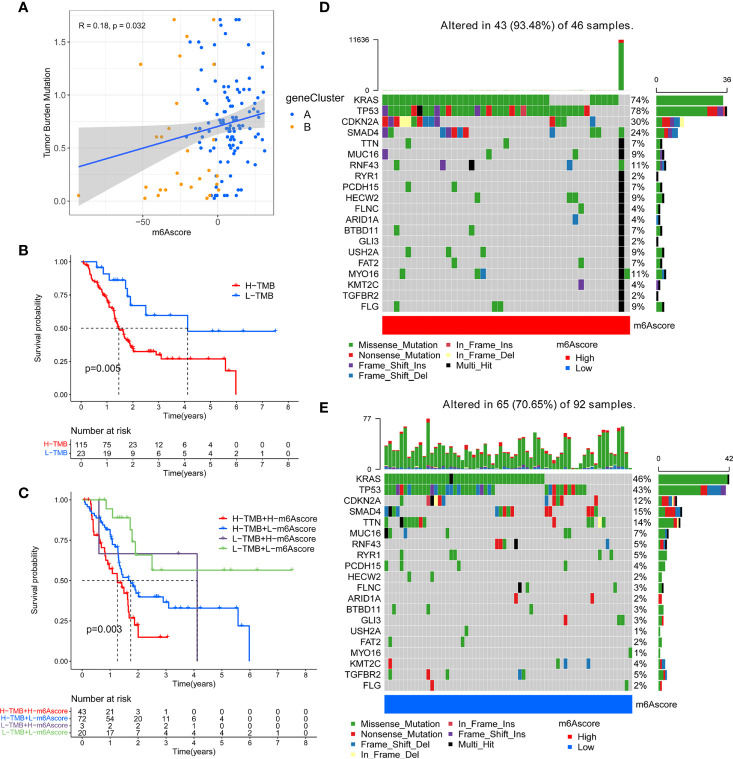
Correlation between m^6^A score and tumor mutation burden (TMB) in TCGA-PAAD cohort. **(A)** A scatter plot describing the positive correlation between m^6^A score and tumor mutation burden. **(B)** Survival analysis of high TMB group and low TMB group. **(C)** Stratified survival analysis including TMB and m^6^A scores. **(D)** OncoPrint for gene mutations in the high m^6^A score group. **(E)** OncoPrint for gene mutations in the low m^6^A score group.

Previous studies have shown that tumor mutation burden may be an emerging and potential tumor marker, which can assist in the selection of patients for immune checkpoint therapy. In view of the important clinical significance of TMB, we tried to explore the inner link between TMB and m^6^A score to clarify the genetic imprint of each m^6^A score subgroup. Correlation analysis showed a significant and positive association between m^6^A score and TBM (Spearman coefficient: R = 0.18, P = 0.032; **Figure 6A**). Next, we divided patients into two subgroups based on TBM. As shown in **Figure 6B**, we found that patients with low TMB showed better overall survival than patients with high TMB. Next, we evaluated the synergy of these scores in the prognostic stratification of PC. Survival analysis demonstrated that TBM status did not affect predictions based on m^6^A score, and the low m^6^A score group always showed a survival advantage (**Figure 6C**). In addition, we analyzed the somatic mutation landscape in the high and low m^6^A score groups, and found that the high m^6^A score group had a higher mutation rate (93.48%) than the low score group (70.65%). According to the results (**Figures 6D, E**), both KRAS (74% *vs.* 46%) and TP53 (78% *vs.* 43%) had a higher somatic mutation rate in the high m^6^A score group, which may be related to the poor prognosis of high m^6^A score group. These data can more comprehensively describe the impact of m^6^A score on genomic variation, and may provide new ideas for studying the potential interaction between m^6^A methylation modification and somatic mutation.

### The Role of m^6^A Score in Predicting the Effect of Immunotherapy

**Figure 7:**
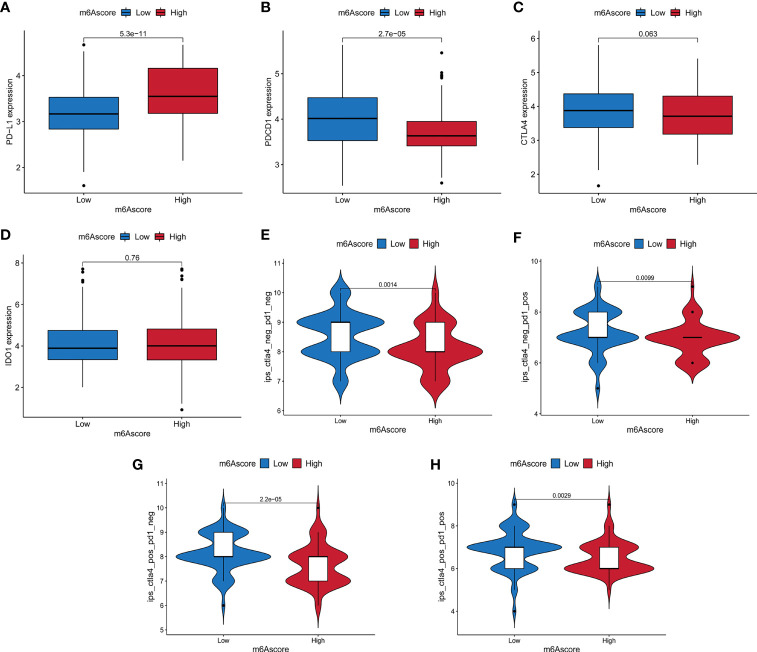
The relationship between m^6^A score and immune checkpoint genes and immunotherapy. **(A–D)** The expression differences of PD-L1, PDCD1, CTLA4 and IDO1 between high and low m^6^A score groups. **(E)** The difference of Immunophenoscore (IPS) between high and low m^6^A score groups with CTLA4 (-)/PD1 (-). **(F)** The difference of IPS between high and low m^6^A score groups with CTLA4 (-)/PD1 (+). **(G)** The difference of IPS between high and low m^6^A score groups with CTLA4 (+)/PD1 (-). **(H)** The difference of IPS between high and low m^6^A score groups with CTLA4 (+)/PD1 (+).

The treatment of immune checkpoint inhibitors represented by CTLA-4/PD-1 inhibitors is undoubtedly a major progress in anti-tumor therapy. We compared the expression of common immune checkpoint genes between the high and low m^6^A score groups, and found that PD-L1 was highly expressed in the high m^6^A score group, PDCD1 was highly expressed in the low m^6^A score group, while the expression of CTLA4 and IDO1 had no significant difference between the two groups (**Figures 7A–D**). As a new predictor of the immune response, IPS is widely used and recommended for evaluating the immune response of patients. Our analysis showed that the IPS of the low m^6^A score group was higher no matter in the case of anti-PD-1/CTLA-4 therapy alone, or combination therapy (**Figures 7E–H**). These results indicated that m^6^A methylation modification in pancreatic cancer may play an important role in mediating the immune response.

## Discussion

Recent studies have shown that m^6^A methylation modification plays an indispensable role in a variety of immune-related biological processes, including innate and acquired immune response, immune recognition, immune cell dynamic balance, and anti-tumor immune response (22). Since most studies mainly focus on the regulatory relationship between single m^6^A regulator and immune cell type, the comprehensive landscape of TME immune-infiltrating mediated by multiple m^6^A regulators in PC has not been fully understood. Therefore, clarifying the characteristics of immune cell infiltration in different m^6^A modification patterns will help us to improve our understanding of the anti-tumor immune response in TME, and provide new insights for the risk stratification of patients and the choice of clinical treatment strategies.

Based on 23 m^6^A regulators, we first identified two m^6^A modification subtypes. Differences in mRNA transcriptomes between different m^6^A modification subtype were found to be closely related to tumor metabolism and immune-related biological processes, and differentially expressed genes (DEGs) were significantly enriched in glucose metabolism-related pathways, chemokine-related pathways, cytokine-related pathways, and TGF-β signaling pathways. DEGs correlated to the prognosis of PC were defined as m^6^A-related signature genes. Based on the m^6^A signature genes, we determined two m^6^A-related gene subtypes. In the two gene clusters, we found that gene cluster A had lower immune score, stromal score and immune response-related T cell infiltration, which suggested an immune cold phenotype. In contrast, gene cluster B showed a relatively high immune score and T cell infiltration, which corresponded to the immune activation phenotype, namely hot tumor. Survival analysis showed that gene cluster A characterized by immune cold phenotype was associated with poor prognosis, while gene cluster B characterized by anti-tumor immune response was associated with good prognosis. We speculate that patients in gene cluster B may benefit from immunotherapy. Our results are consistent with those of previous TME studies, which also indicates that m^6^A methylation modification is of great significance for shaping different TME immune characteristics.

In view of the individual heterogeneity of m^6^A methylation modification, it is necessary to quantify the m^6^A modification pattern of a single tumor sample. Scoring models based on specific biomarkers between m^6^A modified subtypes have been well established in gastric cancer and colorectal cancer to improve the choice of clinical treatment and prognosis of patients (12, 13). Based on principal component analysis and a method similar to GGI, we established an m^6^A scoring scheme for PC patients. Gene cluster B with immune hot phenotype showed lower m^6^A score, while gene cluster A with immune cold phenotype indicated higher m^6^A score. Kaplan-Meier analysis showed that the m^6^A score had good prognostic predictive ability. The survival rate of patients in the low m^6^A score group was higher and the prognosis was better. These results suggest that the m^6^A score is a reliable index to comprehensively evaluate the m^6^A modification pattern of individual tumors, and can be used to further determine the characteristics of TME immune cell infiltration, namely tumor immunophenotype. Besides, verification in the TCGA-PAAD cohort showed that m^6^A score can be used as an independent prognostic indicator for PC patients. And the nomogram constructed by m^6^A score combined with other clinical variables can effectively predict the prognosis of patients.

Evaluation of potential mutation driver genes in tumors is an important means to explore the potential mechanisms of cancer occurrence and development, which is conducive to cancer diagnosis and rational selection of treatment strategies. We found that the mutation rates of KRAS, TP53, CDKN2A, and AMAD4 were significantly increased in the high m^6^A score group. An important feature of KRAS mutant tumors is the immunosuppressive state (40). KRAS signaling induces the expression of immune regulatory factors and inflammatory cytokines in tumor cells, and subsequently recruits neutrophils and myeloid-derived suppressor cells (MDSC) to form an immunosuppressive tumor microenvironment (41). Mutated KRAS in pancreatic cancer plays a central role in tumor development and growth by regulating T-cell cytokines in TME. By acting on downstream effectors, KRAS leads to impaired T-cell recognition of tumor cells, which may mediate immunity escape (22, 42). There are mutations of TP53 in most types of cancers. The deletion or mutation of TP53 in cancers will affect the recruitment and activity of T cells, leading to immune evasion and promoting cancer progression (43, 44). The loss of P53 (encoded by TP53) in pancreatic cancer leads to increased infiltration of regulatory T cells (Tregs) in the peripheral and intratumoral tissues (45). CDKN2A is a multifunctional gene that prevents the cell cycle at the G1/S checkpoint through the CKD4/6 regulatory mechanism. It is reported that approximately 60% of patients with pancreatic ductal adenocarcinoma carry the CDKN2A mutation, and this mutation is associated with a high risk of tumor development (46, 47). SMAD4 is a member of the SMAD family and participates in the transforming growth factor-β (TGF-β) pathway, which inhibits the activity of normal immune cells and promotes the immune escape of cancer cells (48, 49). These studies suggest that KRAS, TP53, CDKN2A, and AMAD4 mutations may be involved in the formation of immune suppression and immune escape in the high m^6^A score group. These m^6^A score-related gene mutations are closely related to the immune activity in TME, indicating that there may be a potential interaction between m^6^A methylation modification and tumor immune genomics. Because the mutation data of pancreatic cancer in the TCGA database is not sufficient, and only a few genes have obvious somatic mutations, it is necessary to verify the mutation oncoprint and explore the underlying mechanism in a larger data set.

In this study, we demonstrated that m^6^A modification patterns played an important role in the formation of different TME immune infiltration landscapes, which suggested that m^6^A methylation modification may affect the therapeutic effect of immune checkpoint inhibitors. We found that the expression of PD-L1 was higher in the high m^6^A score group. Previous studies suggested that pancreatic cancer had an immunosuppressive tumor microenvironment with high PD-L1 expression, which inhibited the cytotoxicity of activated T cells, and PD-L1 overexpression was associated with a poor prognosis (50, 51). We also compared the IPS that predicted the efficacy of anti-PD-1/CTLA-4 regimens in the high and low m^6^A score groups. The low score group had higher IPS, which indicated a relatively better immunotherapy effect. However, our results do not imply causal associations of m6A score and anti-tumor immunity in PC, more clinical evidence needs to be collected in future studies to verify the relationship between m^6^A score and immunotherapy. The above analysis suggests that m^6^A modification characteristics combined with TME status, tumor mutation burden, neoantigen load, PD-L1 expression, IPS and other biomarkers may be a more effective predictive strategy for immunotherapy.

Our research still has some shortcomings. Although we included 23 recognized m^6^A regulators through literature review, newly identified regulators still need to be added into the model to improve the accuracy of the identification of m^6^A methylation modification patterns. In addition, since not all patients with low m^6^A scores can benefit from immunotherapy, more clinicopathological features need to be combined to improve the accuracy of prediction. Although we obtained 434 PC samples from different cohorts, the number of samples may be relatively insufficient, and our findings need to be further validated in a prospective cohort of PC patients receiving immunotherapy.

The study was done within tumor microenvironment in a whole, without distinguishing tumor component, immune component, and stromal component furthermore. This may cause some subtype information to be masked due to the mixture of the component, which is also a shortcoming of our research. We were more concerned about proposing molecular subtypes related to m^6^A methylation in the overall tumor microenvironment and further constructing scores. Subsequent clinical analysis showed that the m^6^A score could be used as an important supplement to existing clinical variables, and could effectively predict the prognosis of patients in combination with other clinical indicators. We may refine the differentiation of the various components of the tumor microenvironment in subsequent research work, and try to use single-cell analysis to distinguish cell types to obtain more information.

This study has provided some new insights into the clinical application of immunotherapy. By targeting m^6^A regulators or m^6^A-related signature genes to change the m^6^A modification pattern and further reverse the poor infiltration of immune cells in TME, that is, the transformation of immune cold tumors to hot tumors, may contribute to the future development of new immunotherapy drugs or combination therapy strategies. In addition, the combination of therapeutic strategies for KRAS, TP53, CDKN2A, and AMAD4 mutations with immunotherapy may open up a new way for the selection of treatment options and reverse the immunosuppressive state in tumors. These findings are conducive to the identification of different immunophenotypes, thereby improving the patient’s response to immunotherapy, and can promote the clinical practice of personalized immunotherapy for cancer.

In conclusion, we evaluated 23 regulators-mediated m^6^A methylation modification landscapes based on 434 PC samples, and correlated m^6^A modification with the TME immune-infiltrating characteristics. And we constructed the m^6^A score, which can comprehensively evaluate the m^6^A modification pattern and immune-infiltrating characteristics of individual tumors, and further determine the tumor immunophenotype to guide clinical application.

## Data Availability Statement

The datasets presented in this study can be found in online repositories. The names of the repository/repositories and accession number(s) can be found in the article/**Supplementary Material**.

## Author Contributions

LX and MS designed the work. MX, TZ, and YW collected and integrated the data. MS analyzed the data and prepared the manuscript. MS, WH, and LX edited and revised the manuscript. All authors contributed to the article and approved the submitted version.

## Funding

This research was funded by National Natural Science Foundation of China No. 81871911 (WH), No. 81972237 (LX), and No. 81772623 (LX), and the National Key Research and Development Program of China 2018YFC1312103 (LX).

## Conflict of Interest

The authors declare that the research was conducted in the absence of any commercial or financial relationships that could be construed as a potential conflict of interest.

## Publisher’s Note

All claims expressed in this article are solely those of the authors and do not necessarily represent those of their affiliated organizations, or those of the publisher, the editors and the reviewers. Any product that may be evaluated in this article, or claim that may be made by its manufacturer, is not guaranteed or endorsed by the publisher.

## Abbreviations

m^6^A, N^6^-methyladenosine; TME, Tumor microenvironment; m^7^G, N^7^-methylguanine; m^5^C, N^5^-methylcytosine; PC, Pancreatic cancer; CAR-T, chimeric antigen receptor T; DC, Dendritic cells; TCGA, The Cancer Genome Atlas; GEO, Gene Expression Ominibus; TPMs, transcripts per kilobase million; CNV, copy number variation; CDF, cumulative distribution function; LM22, Leukocyte signature matrix; DEGs, differentially expressed genes; GO, Gene Ontology; MF, molecular function; BP, biological processes; CC, cellular components; KEGG, Kyoto Encyclopedia of Genes and Genomes; PCA, principal component analysis; ROC, receiver operating characteristic; TMB, tumor mutation burden; IPS, Immunophenoscore; MHC, MHC-related molecules; CP, immunomodulators; EC, effector cells; SC, suppressor cells; TCIA, The Cancer Immunome Atlas; AUC, area under curve; PurIST, purity Independent Subtyping of Tumors; MDSC, myeloid-derived suppressor cells; Tregs, regulatory T cells; TGF-β, transforming growth factor-β.

## References

[1] Zheng HX Zhang XS Sui N. Advances in the Profiling of N(6)-Methyladenosine (M(6)a) Modifications. Biotechnol Adv (2020) 45:107656. doi: 10.1016/j.biotechadv.2020.107656 33181242

[2] Dai F Wu Y Lu Y An C Zheng X Dai L. Crosstalk Between RNA M(6)a Modification and non-Coding RNA Contributes to Cancer Growth and Progression. Mol Ther Nucleic Acids (2020) 22:62–71. doi: 10.1016/j.omtn.2020.08.004 32911345 PMC7486578

[3] Huang H Weng H Chen J. M(6)a Modification in Coding and Non-Coding Rnas: Roles and Therapeutic Implications in Cancer. Cancer Cell (2020) 37:270–88. doi: 10.1016/j.ccell.2020.02.004 PMC7141420 32183948

[4] Zhao W Qi X Liu L Ma S Liu J Wu J. Epigenetic Regulation of M(6)a Modifications in Human Cancer. Mol Ther Nucleic Acids (2020) 19:405–12. doi: 10.1016/j.omtn.2019.11.022 PMC6938965 31887551

[5] Huang H Weng H Chen J. The Biogenesis and Precise Control of RNA M(6)a Methylation. Trends Genet (2020) 36:44–52. doi: 10.1016/j.tig.2019.10.011 31810533 PMC6925345

[6] Yang C Hu Y Zhou B Bao Y Li Z Gong C. The Role of M(6)a Modification in Physiology and Disease. Cell Death Dis (2020) 11:960. doi: 10.1038/s41419-020-03143-z 33162550 PMC7649148

[7] Nombela P Miguel-López B Blanco S. The Role of M(6)a, M(5)C and Ψ RNA Modifications in Cancer: Novel Therapeutic Opportunities. Mol Cancer (2021) 20:18. doi: 10.1186/s12943-020-01263-w 33461542 PMC7812662

[8] Han SH Choe J. Diverse Molecular Functions of M(6)a mRNA Modification in Cancer. Exp Mol Med (2020) 52:738–49. doi: 10.1038/s12276-020-0432-y PMC7272606 32404927

[9] Gu J Zhan Y Zhuo L Zhang Q Li G Li Q. Biological Functions of M(6)a Methyltransferases. Cell Biosci (2021) 11:15. doi: 10.1186/s13578-020-00513-0 33431045 PMC7798219

[10] Jiang X Liu B Nie Z Duan L Xiong Q Jin Z. The Role of M6a Modification in the Biological Functions and Diseases. Signal Transduct Target Ther (2021) 6:74. doi: 10.1038/s41392-020-00450-x 33611339 PMC7897327

[11] Uddin MB Wang Z Yang C. The M(6)a RNA Methylation Regulates Oncogenic Signaling Pathways Driving Cell Malignant Transformation and Carcinogenesis. Mol Cancer (2021) 20:61. doi: 10.1186/s12943-021-01356-0 33814008 PMC8019509

[12] Zhang B Wu Q Li B Wang D Wang L Zhou YL. M(6)a Regulator-Mediated Methylation Modification Patterns and Tumor Microenvironment Infiltration Characterization in Gastric Cancer. Mol Cancer (2020) 19:53. doi: 10.1186/s12943-020-01170-0 32164750 PMC7066851

[13] Chong W Shang L Liu J Fang Z Du F Wu H. M(6)a Regulator-Based Methylation Modification Patterns Characterized by Distinct Tumor Microenvironment Immune Profiles in Colon Cancer. Theranostics (2021) 11:2201–17. doi: 10.7150/thno.52717 PMC7797678 33500720

[14] Mizrahi JD Surana R Valle JW Shroff RT. Pancreatic Cancer. Lancet (2020) 395:2008–20. doi: 10.1016/S0140-6736(20)30974-0 32593337

[15] Klein AP. Pancreatic Cancer Epidemiology: Understanding the Role of Lifestyle and Inherited Risk Factors. Nat Rev Gastroenterol Hepatol (2021) 18:493–502. doi: 10.1038/s41575-021-00457-x 34002083 PMC9265847

[16] Mahajan UM Langhoff E Goni E Costello E Greenhalf W Halloran C. Immune Cell and Stromal Signature Associated With Progression-Free Survival of Patients With Resected Pancreatic Ductal Adenocarcinoma. Gastroenterology (2018) 155:1625–39. doi: 10.1053/j.gastro.2018.08.009 30092175

[17] Fridman WH Zitvogel L Sautès-Fridman C Kroemer G. The Immune Contexture in Cancer Prognosis and Treatment. Nat Rev Clin Oncol (2017) 14:717–34. doi: 10.1038/nrclinonc.2017.101 28741618

[18] Turley SJ Cremasco V Astarita JL. Immunological Hallmarks of Stromal Cells in the Tumour Microenvironment. Nat Rev Immunol (2015) 15:669–82. doi: 10.1038/nri3902 26471778

[19] Ren B Cui M Yang G Wang H Feng M You L. Tumor Microenvironment Participates in Metastasis of Pancreatic Cancer. Mol Cancer (2018) 17:108. doi: 10.1186/s12943-018-0858-1 30060755 PMC6065152

[20] Wang S Li Y Xing C Ding C Zhang H Chen L. Tumor Microenvironment in Chemoresistance, Metastasis and Immunotherapy of Pancreatic Cancer. Am J Cancer Res (2020) 10:1937–53. PMC7407356 32774994

[21] Huang X Ding L Liu X Tong R Ding J Qian Z. Regulation of Tumor Microenvironment for Pancreatic Cancer Therapy. Biomaterials (2021) 270:120680. doi: 10.1016/j.biomaterials.2021.120680 33588140

[22] Torphy RJ Schulick RD Zhu Y. Understanding the Immune Landscape and Tumor Microenvironment of Pancreatic Cancer to Improve Immunotherapy. Mol Carcinog (2020) 59:775–82. doi: 10.1002/mc.23179 32166821

[23] Henze J Tacke F Hardt O Alves F Al RW. Enhancing the Efficacy of CAR T Cells in the Tumor Microenvironment of Pancreatic Cancer. Cancers (Basel) (2020) 12: 1389. doi: 10.3390/cancers12061389 PMC7353070 32481570

[24] Liu Y Liang G Xu H Dong W Dong Z Qiu Z. Tumors Exploit FTO-Mediated Regulation of Glycolytic Metabolism to Evade Immune Surveillance. Cell Metab (2021) 33:1221–33. doi: 10.1016/j.cmet.2021.04.001 33910046

[25] Han D Liu J Chen C Dong L Liu Y Chang R. Anti-Tumour Immunity Controlled Through Mrna M(6)a Methylation and YTHDF1 in Dendritic Cells. Nature (2019) 566:270–4. doi: 10.1038/s41586-019-0916-x PMC6522227 30728504

[26] Wang L Hui H Agrawal K Kang Y Li N Tang R. M(6) a RNA Methyltransferases METTL3/14 Regulate Immune Responses to Anti-PD-1 Therapy. EMBO J (2020) 39:e104514. doi: 10.15252/embj.2020104514 32964498 PMC7560214

[27] Moffitt RA Marayati R Flate EL Volmar KE Loeza SG Hoadley KA. Virtual Microdissection Identifies Distinct Tumor- and Stroma-Specific Subtypes of Pancreatic Ductal Adenocarcinoma. Nat Genet (2015) 47:1168–78. doi: 10.1038/ng.3398 PMC4912058 26343385

[28] Rashid NU Peng XL Jin C Moffitt RA Volmar KE Belt BA. Purity Independent Subtyping of Tumors (Purist), A Clinically Robust, Single-Sample Classifier for Tumor Subtyping in Pancreatic Cancer. Clin Cancer Res (2020) 26:82–92. doi: 10.1158/1078-0432.CCR-19-1467 31754050 PMC6942634

[29] Meng Z Yuan Q Zhao J Wang B Li S Offringa R. The M(6)a-Related Mrna Signature Predicts the Prognosis of Pancreatic Cancer Patients. Mol Ther Oncolytics (2020) 17:460–70. doi: 10.1016/j.omto.2020.04.011 PMC7256444 32490170

[30] Liu H Qiu C Wang B Bing P Tian G Zhang X. Evaluating DNA Methylation, Gene Expression, Somatic Mutation, and Their Combinations in Inferring Tumor Tissue-of-Origin. Front Cell Dev Biol (2021) 9:619330. doi: 10.3389/fcell.2021.619330 34012960 PMC8126648

[31] Șenbabaoğlu Y Michailidis G Li JZ. Critical Limitations of Consensus Clustering in Class Discovery. Sci Rep (2014) 4:6207. doi: 10.1038/srep06207 25158761 PMC4145288

[32] Tibshirani R Walther G Hastie T. Estimating the Number of Clusters in a Data Set via the Gap Statistic. J R Stat Soc Ser B Stat Methodol (2001) 63:411–23. doi: 10.1111/1467-9868.00293

[33] Dudoit S Fridlyand JA. Prediction-Based Resampling Method for Estimating the Number of Clusters in a Dataset. Genome Biol (2002) 3:H36. doi: 10.1186/gb-2002-3-7-research0036 PMC126241 12184810

[34] Wilkerson MD Hayes DN. Consensusclusterplus: A Class Discovery Tool With Confidence Assessments and Item Tracking. Bioinformatics (2010) 26:1572–3. doi: 10.1093/bioinformatics/btq170 PMC2881355 20427518

[35] Newman AM Steen CB Liu CL Gentles AJ Chaudhuri AA Scherer F. Determining Cell Type Abundance and Expression From Bulk Tissues With Digital Cytometry. Nat Biotechnol (2019) 37:773–82. doi: 10.1038/s41587-019-0114-2 PMC6610714 31061481

[36] Yoshihara K Shahmoradgoli M Martínez E Vegesna R Kim H Torres-Garcia W. Inferring Tumour Purity and Stromal and Immune Cell Admixture From Expression Data. Nat Commun (2013) 4:2612. doi: 10.1038/ncomms3612 24113773 PMC3826632

[37] Sotiriou C Wirapati P Loi S Harris A Fox S Smeds J. Gene Expression Profiling in Breast Cancer: Understanding the Molecular Basis of Histologic Grade to Improve Prognosis. J Natl Cancer Inst (2006) 98:262–72. doi: 10.1093/jnci/djj052 16478745

[38] Zeng D Li M Zhou R Zhang J Sun H Shi M. Tumor Microenvironment Characterization in Gastric Cancer Identifies Prognostic and Immunotherapeutically Relevant Gene Signatures. Cancer Immunol Res (2019) 7:737–50. doi: 10.1158/2326-6066.CIR-18-0436 30842092

[39] Charoentong P Finotello F Angelova M Mayer C Efremova M Rieder D. Pan-Cancer Immunogenomic Analyses Reveal Genotype-Immunophenotype Relationships and Predictors of Response to Checkpoint Blockade. Cell Rep (2017) 18:248–62. doi: 10.1016/j.celrep.2016.12.019 28052254

[40] Cullis J Das S Bar-Sagi D. Kras and Tumor Immunity: Friend or Foe? Cold Spring Harb Perspect Med (2018) 8:a031849. doi: 10.1101/cshperspect.a031849 29229670 PMC6120695

[41] Merz V Gaule M Zecchetto C Cavaliere A Casalino S Pesoni C. Targeting KRAS: The Elephant in the Room of Epithelial Cancers. Front Oncol (2021) 11:638360. doi: 10.3389/fonc.2021.638360 33777798 PMC7991835

[42] Dey P Li J Zhang J Chaurasiya S Strom A Wang H. Oncogenic KRAS-Driven Metabolic Reprogramming in Pancreatic Cancer Cells Utilizes Cytokines From the Tumor Microenvironment. Cancer Discov (2020) 10:608–25. doi: 10.1158/2159-8290.CD-19-0297 PMC7125035 32046984

[43] Blagih J Buck MD Vousden KH. P53, Cancer and the Immune Response. J Cell Sci (2020) 133: cs237453. doi: 10.1242/jcs.237453 32144194

[44] Blagih J Zani F Chakravarty P Hennequart M Pilley S Hobor S. Cancer-Specific Loss of P53 Leads to a Modulation of Myeloid and T Cell Responses. Cell Rep (2020) 30:481–96. doi: 10.1016/j.celrep.2019.12.028 PMC6963783 31940491

[45] Bezzi M Seitzer N Ishikawa T Reschke M Chen M Wang G. Diverse Genetic-Driven Immune Landscapes Dictate Tumor Progression Through Distinct Mechanisms. Nat Med (2018) 24:165–75. doi: 10.1038/nm.4463 29309058

[46] Bertoli C Skotheim JM de Bruin RA. Control of Cell Cycle Transcription During G1 and s Phases. Nat Rev Mol Cell Biol (2013) 14:518–28. doi: 10.1038/nrm3629 PMC4569015 23877564

[47] Hu C Hart SN Polley EC Gnanaolivu R Shimelis H Lee KY. Association Between Inherited Germline Mutations in Cancer Predisposition Genes and Risk of Pancreatic Cancer. JAMA (2018) 319:2401–9. doi: 10.1001/jama.2018.6228 PMC6092184 29922827

[48] Robert C. A Decade of Immune-Checkpoint Inhibitors in Cancer Therapy. Nat Commun (2020) 11:3801. doi: 10.1038/s41467-020-17670-y 32732879 PMC7393098

[49] Javadrashid D Baghbanzadeh A Derakhshani A Leone P Silvestris N Racanelli V. Pancreatic Cancer Signaling Pathways, Genetic Alterations, and Tumor Microenvironment: The Barriers Affecting the Method of Treatment. Biomedicines (2021) 9:373. doi: 10.3390/biomedicines9040373 33918146 PMC8067185

[50] Macherla S Laks S Naqash AR Bulumulle A Zervos E Muzaffar M. Emerging Role of Immune Checkpoint Blockade in Pancreatic Cancer. Int J Mol Sci (2018) 19:3505. doi: 10.3390/ijms19113505 PMC6274962 30405053

[51] Jiang Y Li Y Zhu B. T-Cell Exhaustion in the Tumor Microenvironment. Cell Death Dis (2015) 6:e1792. doi: 10.1038/cddis.2015.162 26086965 PMC4669840

