# Supplementary material for: m6A Methylation Modification Patterns and Tumor Microenvironment Infiltration Characterization in Pancreatic Cancer
Source: Front Immunol. 2021 Sep 20;12:739768. doi: 10.3389/fimmu.2021.739768 (PMC8488339; doi:10.3389/fimmu.2021.739768)
Supplement: Supplementary file 1 [file DataSheet_1.docx]

Supplementary Material


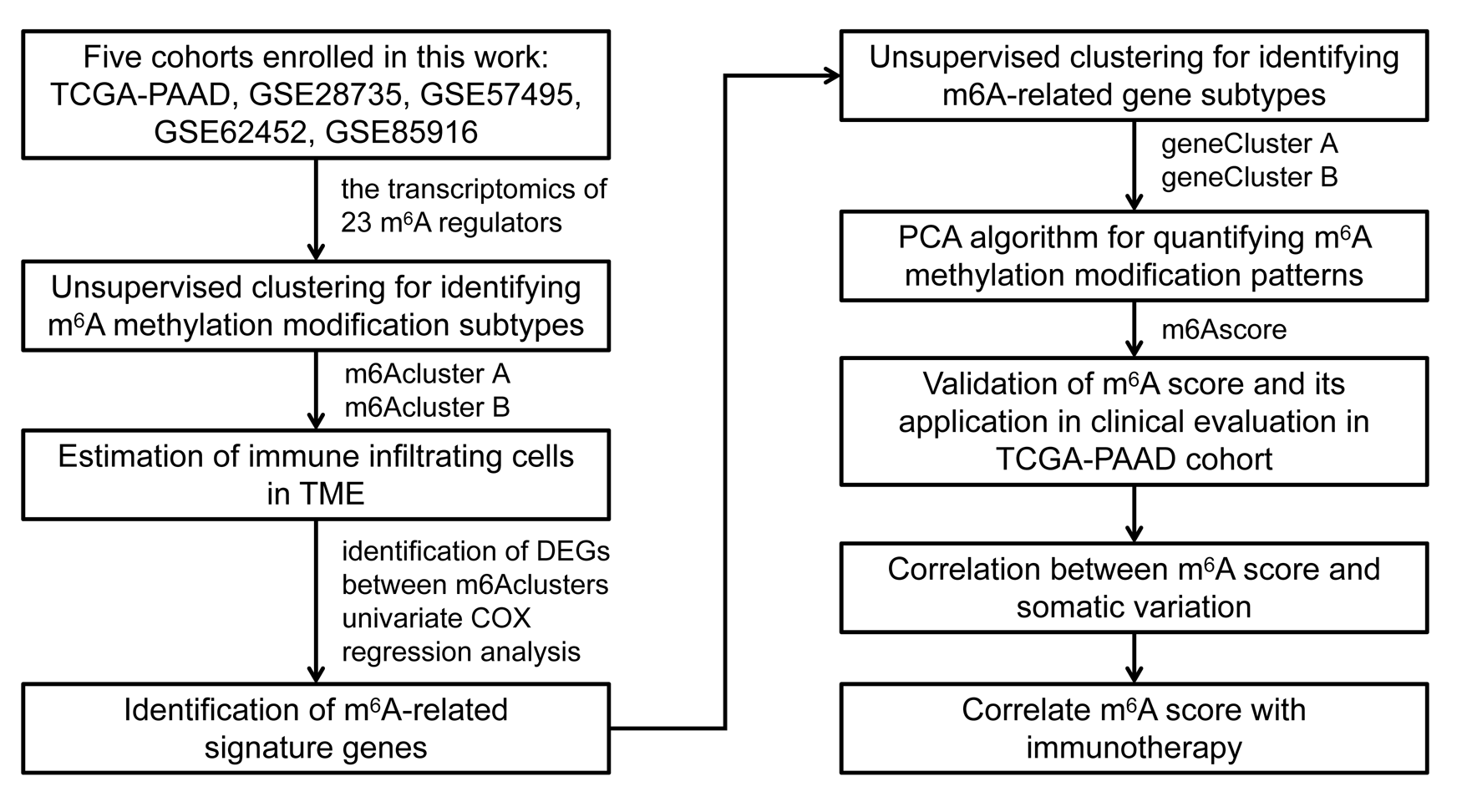


**Supplementary Figure 1.** Overview of study design.


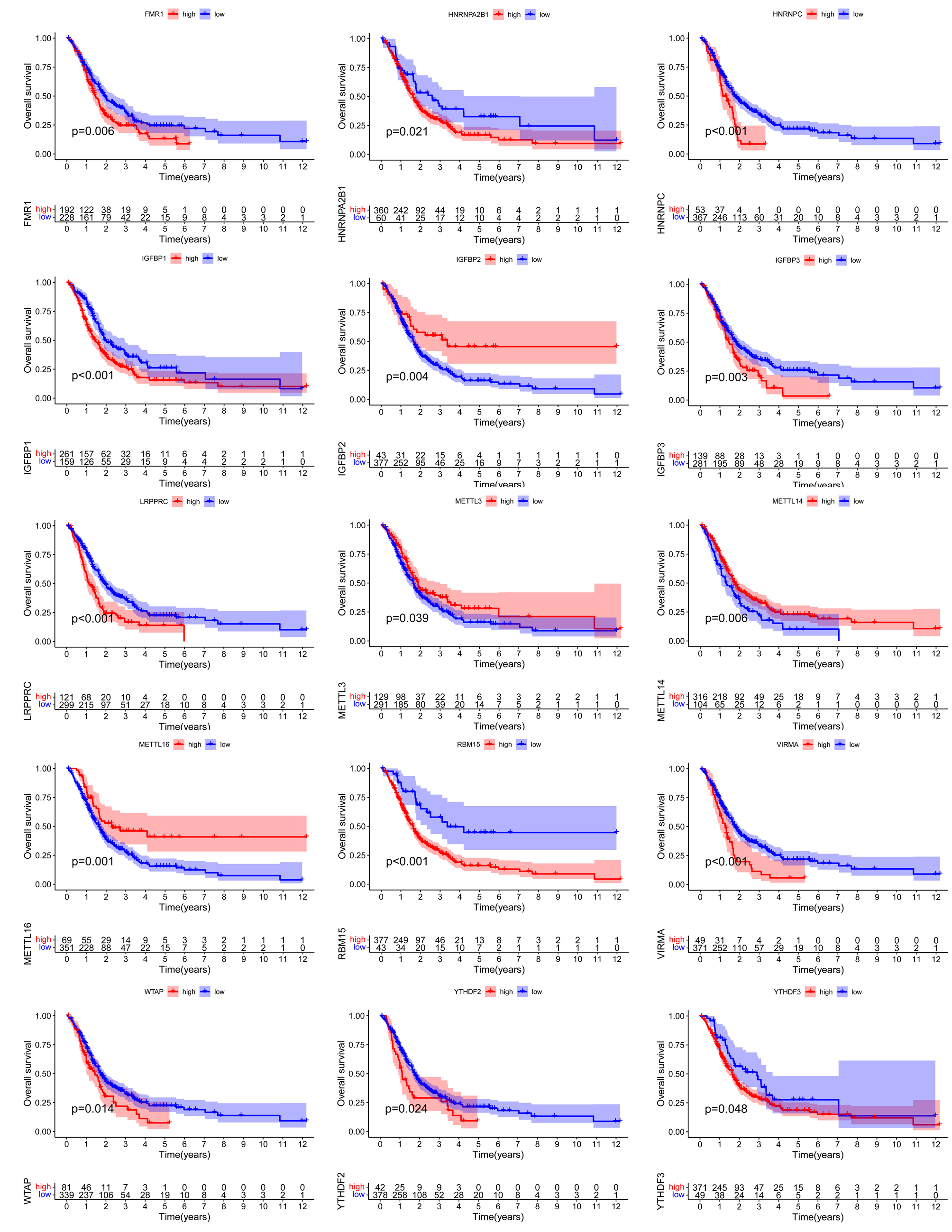


**Supplementary Figure 2.** Kaplan-Meier analysis of 23 m^6^A regulators (only results with significant difference were shown).


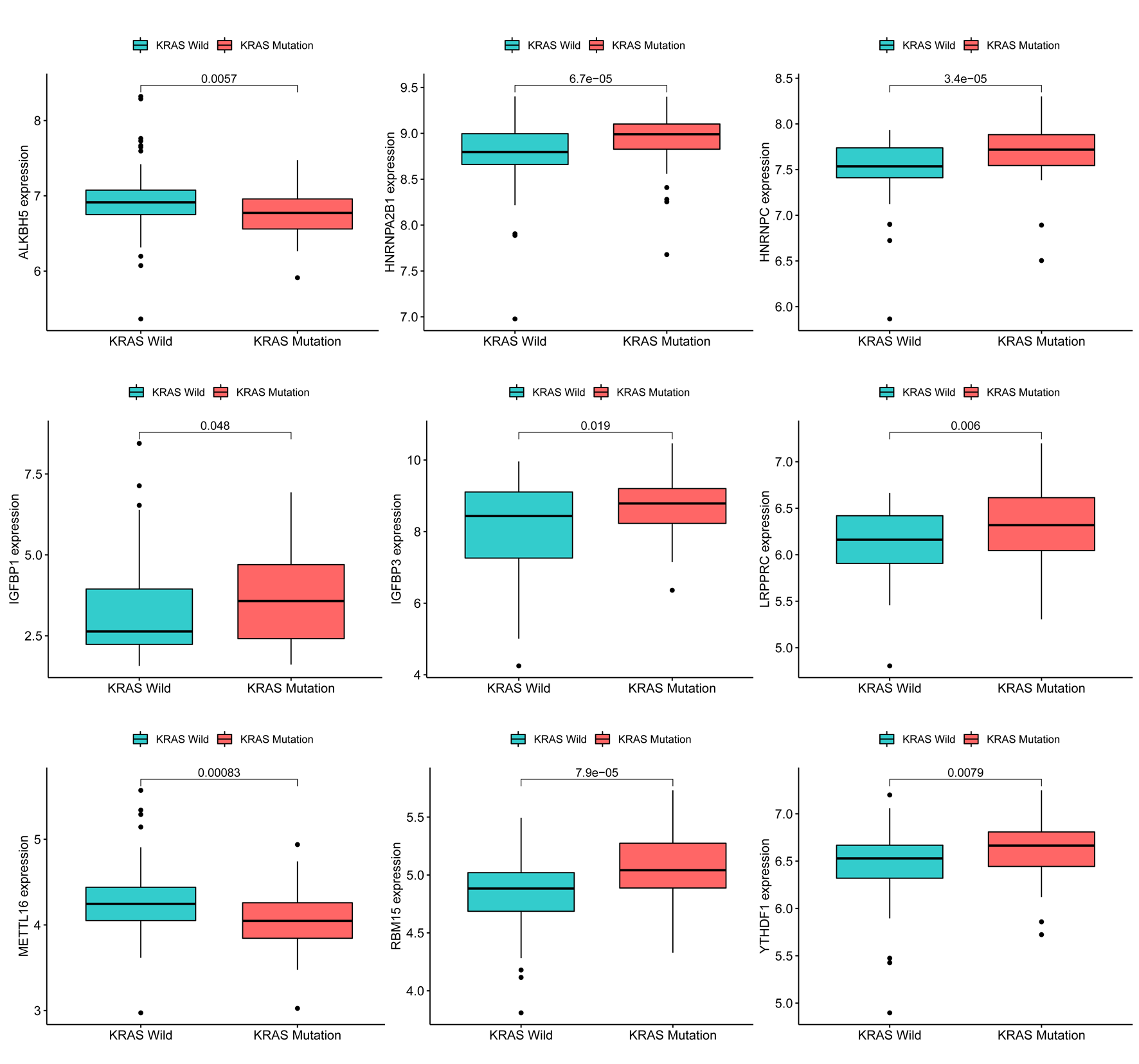


**Supplementary Figure 3.** The expression difference of 23 m6A regulators between wild type and mutant type of KRAS (only results with significant difference were shown).


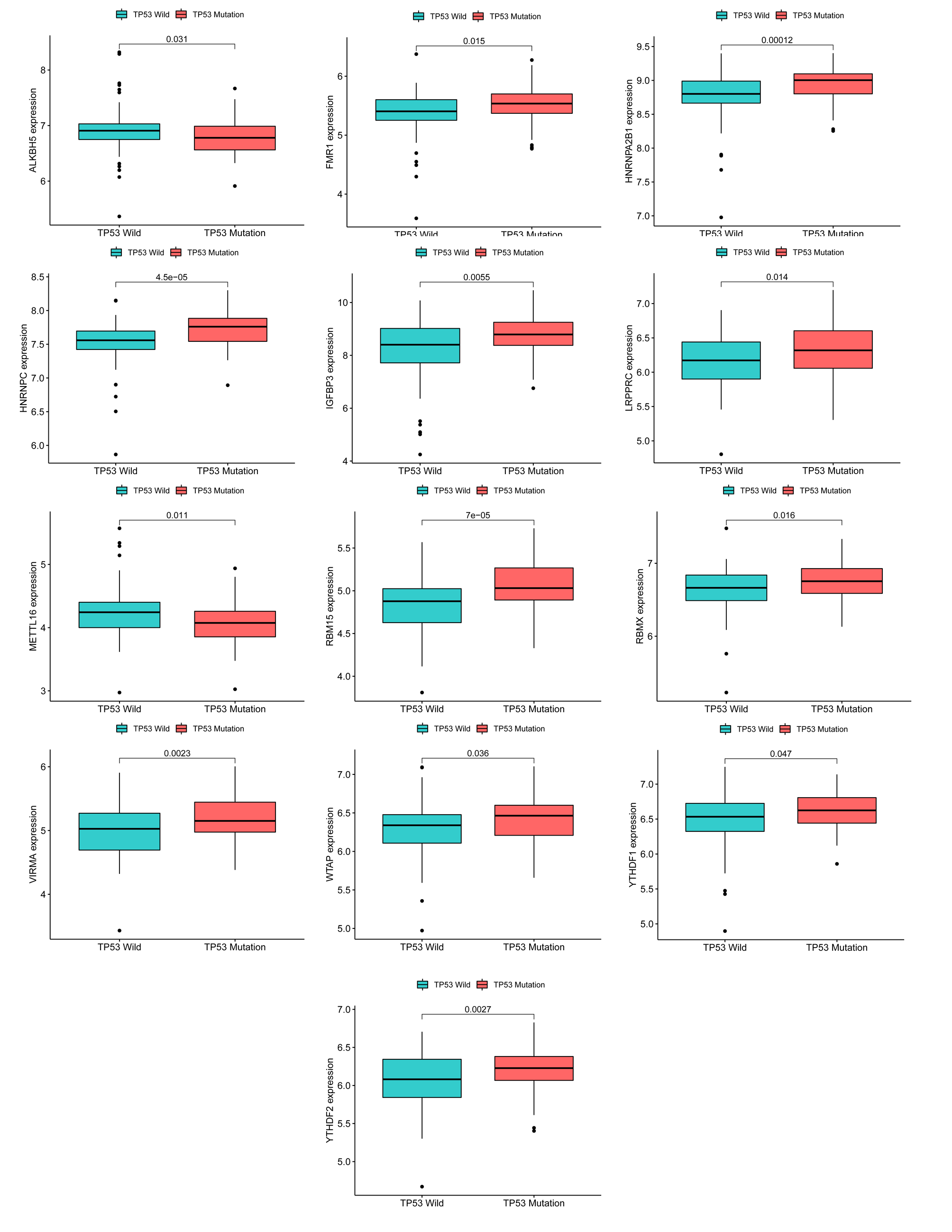


**Supplementary Figure 4.** The expression difference of 23 m6A regulators between wild type and mutant type of TP53 (only results with significant difference were shown).

**Supplementary Table 1.** Prognostic analysis of 21 m6A regulators in pancreatic cancer.

| Gene | HR | HR.95L | HR.95H | pvalue | km |
| --- | --- | --- | --- | --- | --- |
| METTL3 | 0.938596252 | 0.701228793 | 1.256313109 | 0.670103222 | 0.038554357 |
| METTL14 | 0.786581331 | 0.596752111 | 1.036795982 | 0.088467591 | 0.005908646 |
| METTL16 | 0.672245656 | 0.498592472 | 0.906379955 | 0.009196513 | 0.001278252 |
| WTAP | 1.268574859 | 0.895408657 | 1.797260011 | 0.18075992 | 0.014289478 |
| VIRMA | 1.323833095 | 0.953544812 | 1.837914739 | 0.093776762 | 0.00064614 |
| ZC3H13 | 1.132859114 | 0.868722271 | 1.477307324 | 0.357067762 | 0.092303498 |
| RBM15 | 1.360434865 | 0.969063828 | 1.909866995 | 0.075338023 | 0.000197323 |
| RBM15B | 0.901035498 | 0.640967133 | 1.266624958 | 0.548683963 | 0.143562233 |
| YTHDC1 | 0.970833418 | 0.680801249 | 1.384423908 | 0.870142363 | 0.053201312 |
| YTHDC2 | 0.977814056 | 0.740424302 | 1.291314081 | 0.874360226 | 0.085011381 |
| YTHDF1 | 1.307412414 | 0.923892564 | 1.850136356 | 0.130250135 | 0.078706109 |
| YTHDF2 | 1.257958636 | 0.883472176 | 1.791182532 | 0.203085722 | 0.023526576 |
| YTHDF3 | 1.161184273 | 0.905202352 | 1.48955525 | 0.23954658 | 0.047708099 |
| HNRNPC | 1.649817937 | 1.118426109 | 2.433687127 | 0.011594375 | 0.000233709 |
| FMR1 | 1.468544923 | 1.042159482 | 2.069380194 | 0.028095754 | 0.006197559 |
| LRPPRC | 1.725955342 | 1.255971404 | 2.37180706 | 0.000764798 | 7.61E-06 |
| HNRNPA2B1 | 1.290070942 | 0.917818939 | 1.813302128 | 0.142572914 | 0.020774366 |
| IGFBP1 | 1.110167063 | 1.022805139 | 1.204990922 | 0.012448003 | 0.000877683 |
| IGFBP2 | 0.866481875 | 0.776715488 | 0.966622722 | 0.01021906 | 0.003751837 |
| IGFBP3 | 1.196782723 | 1.056287225 | 1.355965359 | 0.004810832 | 0.002761768 |
| RBMX | 1.028216234 | 0.706758089 | 1.495884716 | 0.884336916 | 0.21751762 |
| FTO | 1.082880162 | 0.802440081 | 1.461329605 | 0.602586648 | 0.085881866 |
| ALKBH5 | 0.872570103 | 0.635963798 | 1.197204287 | 0.398300601 | 0.184704113 |

**Supplementary Table 2.** 23 regulators-mediated m^6^A methylation modification subtypes

| ID | m6Acluster |
| --- | --- |
| TCGA_TCGA-FB-AAQ1 | A |
| TCGA_TCGA-IB-7888 | A |
| TCGA_TCGA-2J-AABO | A |
| TCGA_TCGA-FB-A5VM | A |
| TCGA_TCGA-HZ-8315 | A |
| TCGA_TCGA-IB-7893 | B |
| TCGA_TCGA-2L-AAQE | A |
| TCGA_TCGA-HZ-8003 | A |
| TCGA_TCGA-YH-A8SY | A |
| TCGA_TCGA-M8-A5N4 | B |
| TCGA_TCGA-2J-AABT | B |
| TCGA_TCGA-IB-AAUU | B |
| TCGA_TCGA-PZ-A5RE | B |
| TCGA_TCGA-HV-A7OL | A |
| TCGA_TCGA-IB-7897 | A |
| TCGA_TCGA-H6-A45N | A |
| TCGA_TCGA-3A-A9IX | B |
| TCGA_TCGA-XD-AAUH | A |
| TCGA_TCGA-HZ-A77O | A |
| TCGA_TCGA-IB-A5SO | B |
| TCGA_TCGA-F2-6879 | A |
| TCGA_TCGA-3A-A9IV | A |
| TCGA_TCGA-HZ-8519 | A |
| TCGA_TCGA-3A-A9IR | A |
| TCGA_TCGA-HV-AA8V | A |
| TCGA_TCGA-Q3-A5QY | A |
| TCGA_TCGA-HZ-A9TJ | A |
| TCGA_TCGA-3A-A9IL | A |
| TCGA_TCGA-H6-8124 | B |
| TCGA_TCGA-3E-AAAY | B |
| TCGA_TCGA-LB-A8F3 | B |
| TCGA_TCGA-US-A77E | A |
| TCGA_TCGA-YY-A8LH | B |
| TCGA_TCGA-IB-7654 | A |
| TCGA_TCGA-FB-A78T | A |
| TCGA_TCGA-2J-AAB8 | B |
| TCGA_TCGA-FB-A4P6 | A |
| TCGA_TCGA-3A-A9I5 | B |
| TCGA_TCGA-HZ-8005 | A |
| TCGA_TCGA-2J-AAB9 | B |
| TCGA_TCGA-XN-A8T5 | A |
| TCGA_TCGA-HZ-8638 | B |
| TCGA_TCGA-L1-A7W4 | A |
| TCGA_TCGA-US-A779 | B |
| TCGA_TCGA-HZ-7925 | B |
| TCGA_TCGA-IB-7886 | A |
| TCGA_TCGA-IB-8127 | A |
| TCGA_TCGA-XN-A8T3 | A |
| TCGA_TCGA-FB-AAQ0 | A |
| TCGA_TCGA-S4-A8RM | A |
| TCGA_TCGA-IB-AAUP | A |
| TCGA_TCGA-HZ-8317 | B |
| TCGA_TCGA-3A-A9I9 | A |
| TCGA_TCGA-F2-6880 | A |
| TCGA_TCGA-2L-AAQJ | A |
| TCGA_TCGA-3A-A9IH | B |
| TCGA_TCGA-IB-A5ST | A |
| TCGA_TCGA-3A-A9IU | A |
| TCGA_TCGA-HV-AA8X | B |
| TCGA_TCGA-HZ-A77Q | B |
| TCGA_TCGA-HZ-7924 | A |
| TCGA_TCGA-HZ-7289 | A |
| TCGA_TCGA-HZ-7926 | A |
| TCGA_TCGA-RB-AA9M | B |
| TCGA_TCGA-3A-A9IO | A |
| TCGA_TCGA-2J-AABH | A |
| TCGA_TCGA-IB-7645 | A |
| TCGA_TCGA-2J-AABA | A |
| TCGA_TCGA-HZ-A4BH | A |
| TCGA_TCGA-Q3-AA2A | B |
| TCGA_TCGA-FB-AAQ2 | B |
| TCGA_TCGA-2L-AAQM | A |
| TCGA_TCGA-IB-7649 | A |
| TCGA_TCGA-LB-A9Q5 | B |
| TCGA_TCGA-HZ-A8P0 | A |
| TCGA_TCGA-S4-A8RO | B |
| TCGA_TCGA-3A-A9IC | A |
| TCGA_TCGA-IB-AAUN | A |
| TCGA_TCGA-HZ-A49G | A |
| TCGA_TCGA-S4-A8RP | B |
| TCGA_TCGA-HZ-A49H | A |
| TCGA_TCGA-IB-AAUM | A |
| TCGA_TCGA-HZ-A8P1 | A |
| TCGA_TCGA-2J-AABV | A |
| TCGA_TCGA-IB-A5SS | B |
| TCGA_TCGA-FB-AAPZ | B |
| TCGA_TCGA-2J-AABI | A |
| TCGA_TCGA-IB-AAUO | B |
| TCGA_TCGA-2J-AAB6 | A |
| TCGA_TCGA-2J-AABF | A |
| TCGA_TCGA-HZ-8002 | A |
| TCGA_TCGA-FB-AAQ6 | B |
| TCGA_TCGA-2J-AAB1 | A |
| TCGA_TCGA-HV-A5A6 | A |
| TCGA_TCGA-HZ-8001 | A |
| TCGA_TCGA-3E-AAAZ | B |
| TCGA_TCGA-IB-7885 | B |
| TCGA_TCGA-IB-7651 | B |
| TCGA_TCGA-HZ-7923 | A |
| TCGA_TCGA-HZ-8637 | A |
| TCGA_TCGA-HZ-A77P | A |
| TCGA_TCGA-IB-7646 | A |
| TCGA_TCGA-HV-A5A3 | A |
| TCGA_TCGA-HZ-7920 | B |
| TCGA_TCGA-FB-AAPU | A |
| TCGA_TCGA-HZ-7922 | B |
| TCGA_TCGA-IB-A6UF | B |
| TCGA_TCGA-OE-A75W | A |
| TCGA_TCGA-3A-A9IZ | B |
| TCGA_TCGA-3A-A9IN | A |
| TCGA_TCGA-F2-7276 | B |
| TCGA_TCGA-HV-A5A5 | A |
| TCGA_TCGA-2J-AABU | A |
| TCGA_TCGA-US-A77J | B |
| TCGA_TCGA-2J-AABE | B |
| TCGA_TCGA-RB-A7B8 | A |
| TCGA_TCGA-IB-A6UG | A |
| TCGA_TCGA-3A-A9J0 | A |
| TCGA_TCGA-IB-AAUQ | B |
| TCGA_TCGA-FB-A7DR | B |
| TCGA_TCGA-2L-AAQL | B |
| TCGA_TCGA-HZ-7918 | A |
| TCGA_TCGA-IB-7887 | A |
| TCGA_TCGA-2J-AABR | B |
| TCGA_TCGA-IB-A5SP | A |
| TCGA_TCGA-3A-A9I7 | B |
| TCGA_TCGA-FB-AAPP | A |
| TCGA_TCGA-F2-A7TX | A |
| TCGA_TCGA-XD-AAUL | B |
| TCGA_TCGA-F2-A44H | A |
| TCGA_TCGA-IB-7644 | A |
| TCGA_TCGA-3A-A9IB | B |
| TCGA_TCGA-IB-AAUW | A |
| TCGA_TCGA-HZ-7919 | A |
| TCGA_TCGA-F2-7273 | B |
| TCGA_TCGA-2L-AAQI | A |
| TCGA_TCGA-XD-AAUG | A |
| TCGA_TCGA-3A-A9IS | B |
| TCGA_TCGA-IB-AAUT | A |
| TCGA_TCGA-Z5-AAPL | A |
| TCGA_TCGA-HZ-A4BK | A |
| TCGA_TCGA-US-A776 | A |
| TCGA_TCGA-IB-7889 | A |
| TCGA_TCGA-IB-AAUR | A |
| TCGA_TCGA-IB-A7M4 | A |
| TCGA_TCGA-FB-AAPS | A |
| TCGA_TCGA-LB-A7SX | A |
| TCGA_TCGA-IB-AAUS | A |
| TCGA_TCGA-HV-A7OP | B |
| TCGA_TCGA-IB-7652 | A |
| TCGA_TCGA-2J-AAB4 | B |
| TCGA_TCGA-US-A774 | A |
| TCGA_TCGA-IB-8126 | A |
| TCGA_TCGA-F2-A44G | B |
| TCGA_TCGA-IB-AAUV | A |
| TCGA_TCGA-FB-AAQ3 | B |
| TCGA_TCGA-RL-AAAS | A |
| TCGA_TCGA-IB-7890 | A |
| TCGA_TCGA-XD-AAUI | A |
| TCGA_TCGA-2J-AABK | A |
| TCGA_TCGA-FB-A545 | B |
| TCGA_TCGA-3A-A9IJ | A |
| TCGA_TCGA-F2-A8YN | B |
| TCGA_TCGA-HZ-A49I | A |
| TCGA_TCGA-HV-A5A4 | A |
| TCGA_TCGA-HZ-8636 | B |
| TCGA_TCGA-FB-AAPY | A |
| TCGA_TCGA-FB-A4P5 | A |
| TCGA_TCGA-2L-AAQA | B |
| TCGA_TCGA-H8-A6C1 | B |
| TCGA_TCGA-2J-AABP | A |
| TCGA_TCGA-US-A77G | A |
| TCGA_TCGA-IB-A7LX | B |
| TCGA_TCGA-IB-7891 | A |
| TCGA_TCGA-YB-A89D | B |
| TCGA_TCGA-IB-A5SQ | B |
| TCGA_TCGA-FB-AAPQ | A |
| GSE28735geoMatrix_GSM711904 | B |
| GSE28735geoMatrix_GSM711906 | B |
| GSE28735geoMatrix_GSM711908 | B |
| GSE28735geoMatrix_GSM711910 | B |
| GSE28735geoMatrix_GSM711912 | B |
| GSE28735geoMatrix_GSM711914 | A |
| GSE28735geoMatrix_GSM711916 | A |
| GSE28735geoMatrix_GSM711918 | B |
| GSE28735geoMatrix_GSM711920 | A |
| GSE28735geoMatrix_GSM711922 | A |
| GSE28735geoMatrix_GSM711924 | A |
| GSE28735geoMatrix_GSM711926 | A |
| GSE28735geoMatrix_GSM711928 | A |
| GSE28735geoMatrix_GSM711930 | A |
| GSE28735geoMatrix_GSM711932 | B |
| GSE28735geoMatrix_GSM711934 | A |
| GSE28735geoMatrix_GSM711936 | B |
| GSE28735geoMatrix_GSM711938 | A |
| GSE28735geoMatrix_GSM711940 | A |
| GSE28735geoMatrix_GSM711942 | A |
| GSE28735geoMatrix_GSM711944 | A |
| GSE28735geoMatrix_GSM711946 | A |
| GSE28735geoMatrix_GSM711948 | A |
| GSE28735geoMatrix_GSM711950 | A |
| GSE28735geoMatrix_GSM711952 | A |
| GSE28735geoMatrix_GSM711954 | A |
| GSE28735geoMatrix_GSM711956 | B |
| GSE28735geoMatrix_GSM711958 | B |
| GSE28735geoMatrix_GSM711960 | A |
| GSE28735geoMatrix_GSM711962 | A |
| GSE28735geoMatrix_GSM711964 | B |
| GSE28735geoMatrix_GSM711966 | A |
| GSE28735geoMatrix_GSM711968 | A |
| GSE28735geoMatrix_GSM711970 | B |
| GSE28735geoMatrix_GSM711972 | A |
| GSE28735geoMatrix_GSM711974 | B |
| GSE28735geoMatrix_GSM711976 | A |
| GSE28735geoMatrix_GSM711978 | A |
| GSE28735geoMatrix_GSM711980 | A |
| GSE28735geoMatrix_GSM711982 | A |
| GSE28735geoMatrix_GSM711984 | A |
| GSE28735geoMatrix_GSM711986 | A |
| GSE28735geoMatrix_GSM711988 | A |
| GSE28735geoMatrix_GSM711990 | A |
| GSE28735geoMatrix_GSM711992 | B |
| GSE57495geoMatrix_GSM1383778 | B |
| GSE57495geoMatrix_GSM1383779 | A |
| GSE57495geoMatrix_GSM1383780 | B |
| GSE57495geoMatrix_GSM1383781 | B |
| GSE57495geoMatrix_GSM1383782 | B |
| GSE57495geoMatrix_GSM1383783 | A |
| GSE57495geoMatrix_GSM1383784 | B |
| GSE57495geoMatrix_GSM1383785 | B |
| GSE57495geoMatrix_GSM1383786 | A |
| GSE57495geoMatrix_GSM1383787 | A |
| GSE57495geoMatrix_GSM1383788 | A |
| GSE57495geoMatrix_GSM1383789 | A |
| GSE57495geoMatrix_GSM1383790 | A |
| GSE57495geoMatrix_GSM1383791 | A |
| GSE57495geoMatrix_GSM1383792 | A |
| GSE57495geoMatrix_GSM1383793 | A |
| GSE57495geoMatrix_GSM1383794 | A |
| GSE57495geoMatrix_GSM1383795 | B |
| GSE57495geoMatrix_GSM1383796 | A |
| GSE57495geoMatrix_GSM1383797 | B |
| GSE57495geoMatrix_GSM1383798 | A |
| GSE57495geoMatrix_GSM1383799 | A |
| GSE57495geoMatrix_GSM1383800 | B |
| GSE57495geoMatrix_GSM1383801 | B |
| GSE57495geoMatrix_GSM1383802 | B |
| GSE57495geoMatrix_GSM1383803 | B |
| GSE57495geoMatrix_GSM1383804 | A |
| GSE57495geoMatrix_GSM1383805 | B |
| GSE57495geoMatrix_GSM1383806 | B |
| GSE57495geoMatrix_GSM1383807 | B |
| GSE57495geoMatrix_GSM1383808 | A |
| GSE57495geoMatrix_GSM1383809 | A |
| GSE57495geoMatrix_GSM1383810 | A |
| GSE57495geoMatrix_GSM1383811 | B |
| GSE57495geoMatrix_GSM1383812 | A |
| GSE57495geoMatrix_GSM1383813 | A |
| GSE57495geoMatrix_GSM1383814 | A |
| GSE57495geoMatrix_GSM1383815 | A |
| GSE57495geoMatrix_GSM1383816 | A |
| GSE57495geoMatrix_GSM1383817 | A |
| GSE57495geoMatrix_GSM1383818 | B |
| GSE57495geoMatrix_GSM1383819 | A |
| GSE57495geoMatrix_GSM1383820 | B |
| GSE57495geoMatrix_GSM1383821 | A |
| GSE57495geoMatrix_GSM1383822 | A |
| GSE57495geoMatrix_GSM1383823 | B |
| GSE57495geoMatrix_GSM1383824 | A |
| GSE57495geoMatrix_GSM1383825 | A |
| GSE57495geoMatrix_GSM1383826 | A |
| GSE57495geoMatrix_GSM1383827 | B |
| GSE57495geoMatrix_GSM1383828 | B |
| GSE57495geoMatrix_GSM1383829 | A |
| GSE57495geoMatrix_GSM1383830 | B |
| GSE57495geoMatrix_GSM1383831 | A |
| GSE57495geoMatrix_GSM1383832 | B |
| GSE57495geoMatrix_GSM1383833 | A |
| GSE57495geoMatrix_GSM1383834 | A |
| GSE57495geoMatrix_GSM1383835 | A |
| GSE57495geoMatrix_GSM1383836 | A |
| GSE57495geoMatrix_GSM1383837 | B |
| GSE57495geoMatrix_GSM1383838 | A |
| GSE57495geoMatrix_GSM1383839 | B |
| GSE57495geoMatrix_GSM1383840 | B |
| GSE62452geoMatrix_GSM1527105 | B |
| GSE62452geoMatrix_GSM1527107 | B |
| GSE62452geoMatrix_GSM1527109 | B |
| GSE62452geoMatrix_GSM1527111 | B |
| GSE62452geoMatrix_GSM1527113 | B |
| GSE62452geoMatrix_GSM1527115 | A |
| GSE62452geoMatrix_GSM1527117 | A |
| GSE62452geoMatrix_GSM1527119 | B |
| GSE62452geoMatrix_GSM1527121 | A |
| GSE62452geoMatrix_GSM1527123 | A |
| GSE62452geoMatrix_GSM1527125 | A |
| GSE62452geoMatrix_GSM1527127 | A |
| GSE62452geoMatrix_GSM1527129 | A |
| GSE62452geoMatrix_GSM1527131 | B |
| GSE62452geoMatrix_GSM1527133 | B |
| GSE62452geoMatrix_GSM1527135 | A |
| GSE62452geoMatrix_GSM1527137 | B |
| GSE62452geoMatrix_GSM1527139 | A |
| GSE62452geoMatrix_GSM1527141 | A |
| GSE62452geoMatrix_GSM1527143 | A |
| GSE62452geoMatrix_GSM1527145 | A |
| GSE62452geoMatrix_GSM1527147 | A |
| GSE62452geoMatrix_GSM1527149 | A |
| GSE62452geoMatrix_GSM1527151 | A |
| GSE62452geoMatrix_GSM1527153 | A |
| GSE62452geoMatrix_GSM1527155 | B |
| GSE62452geoMatrix_GSM1527157 | B |
| GSE62452geoMatrix_GSM1527159 | B |
| GSE62452geoMatrix_GSM1527161 | A |
| GSE62452geoMatrix_GSM1527163 | A |
| GSE62452geoMatrix_GSM1527165 | B |
| GSE62452geoMatrix_GSM1527167 | A |
| GSE62452geoMatrix_GSM1527169 | A |
| GSE62452geoMatrix_GSM1527171 | B |
| GSE62452geoMatrix_GSM1527173 | A |
| GSE62452geoMatrix_GSM1527175 | B |
| GSE62452geoMatrix_GSM1527177 | A |
| GSE62452geoMatrix_GSM1527179 | A |
| GSE62452geoMatrix_GSM1527181 | A |
| GSE62452geoMatrix_GSM1527183 | A |
| GSE62452geoMatrix_GSM1527185 | A |
| GSE62452geoMatrix_GSM1527187 | A |
| GSE62452geoMatrix_GSM1527189 | A |
| GSE62452geoMatrix_GSM1527191 | A |
| GSE62452geoMatrix_GSM1527193 | B |
| GSE62452geoMatrix_GSM1527196 | B |
| GSE62452geoMatrix_GSM1527198 | A |
| GSE62452geoMatrix_GSM1527199 | A |
| GSE62452geoMatrix_GSM1527200 | A |
| GSE62452geoMatrix_GSM1527202 | A |
| GSE62452geoMatrix_GSM1527204 | B |
| GSE62452geoMatrix_GSM1527205 | A |
| GSE62452geoMatrix_GSM1527207 | A |
| GSE62452geoMatrix_GSM1527209 | A |
| GSE62452geoMatrix_GSM1527210 | A |
| GSE62452geoMatrix_GSM1527212 | A |
| GSE62452geoMatrix_GSM1527213 | A |
| GSE62452geoMatrix_GSM1527215 | A |
| GSE62452geoMatrix_GSM1527216 | A |
| GSE62452geoMatrix_GSM1527218 | A |
| GSE62452geoMatrix_GSM1527219 | B |
| GSE62452geoMatrix_GSM1527220 | B |
| GSE62452geoMatrix_GSM1527223 | A |
| GSE62452geoMatrix_GSM1527225 | B |
| GSE62452geoMatrix_GSM1527227 | B |
| GSE62452geoMatrix_GSM1527228 | A |
| GSE62452geoMatrix_GSM1527230 | A |
| GSE62452geoMatrix_GSM1527232 | A |
| GSE62452geoMatrix_GSM1527234 | A |
| GSE85916geoMatrix_GSM2287304 | A |
| GSE85916geoMatrix_GSM2287305 | A |
| GSE85916geoMatrix_GSM2287306 | A |
| GSE85916geoMatrix_GSM2287307 | B |
| GSE85916geoMatrix_GSM2287308 | A |
| GSE85916geoMatrix_GSM2287309 | A |
| GSE85916geoMatrix_GSM2287310 | A |
| GSE85916geoMatrix_GSM2287311 | A |
| GSE85916geoMatrix_GSM2287312 | A |
| GSE85916geoMatrix_GSM2287313 | A |
| GSE85916geoMatrix_GSM2287314 | A |
| GSE85916geoMatrix_GSM2287315 | A |
| GSE85916geoMatrix_GSM2287316 | A |
| GSE85916geoMatrix_GSM2287317 | A |
| GSE85916geoMatrix_GSM2287318 | A |
| GSE85916geoMatrix_GSM2287319 | A |
| GSE85916geoMatrix_GSM2287320 | A |
| GSE85916geoMatrix_GSM2287321 | A |
| GSE85916geoMatrix_GSM2287322 | B |
| GSE85916geoMatrix_GSM2287323 | B |
| GSE85916geoMatrix_GSM2287324 | B |
| GSE85916geoMatrix_GSM2287325 | A |
| GSE85916geoMatrix_GSM2287326 | A |
| GSE85916geoMatrix_GSM2287327 | A |
| GSE85916geoMatrix_GSM2287328 | A |
| GSE85916geoMatrix_GSM2287329 | A |
| GSE85916geoMatrix_GSM2287330 | B |
| GSE85916geoMatrix_GSM2287331 | A |
| GSE85916geoMatrix_GSM2287332 | A |
| GSE85916geoMatrix_GSM2287333 | A |
| GSE85916geoMatrix_GSM2287334 | A |
| GSE85916geoMatrix_GSM2287335 | A |
| GSE85916geoMatrix_GSM2287336 | A |
| GSE85916geoMatrix_GSM2287337 | A |
| GSE85916geoMatrix_GSM2287338 | A |
| GSE85916geoMatrix_GSM2287339 | A |
| GSE85916geoMatrix_GSM2287340 | A |
| GSE85916geoMatrix_GSM2287341 | A |
| GSE85916geoMatrix_GSM2287342 | A |
| GSE85916geoMatrix_GSM2287343 | A |
| GSE85916geoMatrix_GSM2287344 | A |
| GSE85916geoMatrix_GSM2287345 | A |
| GSE85916geoMatrix_GSM2287346 | A |
| GSE85916geoMatrix_GSM2287347 | B |
| GSE85916geoMatrix_GSM2287348 | B |
| GSE85916geoMatrix_GSM2287349 | A |
| GSE85916geoMatrix_GSM2287350 | A |
| GSE85916geoMatrix_GSM2287351 | A |
| GSE85916geoMatrix_GSM2287352 | B |
| GSE85916geoMatrix_GSM2287353 | A |
| GSE85916geoMatrix_GSM2287354 | A |
| GSE85916geoMatrix_GSM2287355 | A |
| GSE85916geoMatrix_GSM2287356 | A |
| GSE85916geoMatrix_GSM2287357 | B |
| GSE85916geoMatrix_GSM2287358 | A |
| GSE85916geoMatrix_GSM2287359 | A |
| GSE85916geoMatrix_GSM2287360 | B |
| GSE85916geoMatrix_GSM2287361 | A |
| GSE85916geoMatrix_GSM2287362 | A |
| GSE85916geoMatrix_GSM2287363 | A |
| GSE85916geoMatrix_GSM2287364 | A |
| GSE85916geoMatrix_GSM2287365 | A |
| GSE85916geoMatrix_GSM2287366 | B |
| GSE85916geoMatrix_GSM2287367 | B |
| GSE85916geoMatrix_GSM2287368 | A |
| GSE85916geoMatrix_GSM2287369 | A |
| GSE85916geoMatrix_GSM2287370 | A |
| GSE85916geoMatrix_GSM2287371 | B |
| GSE85916geoMatrix_GSM2287372 | A |
| GSE85916geoMatrix_GSM2287373 | A |
| GSE85916geoMatrix_GSM2287374 | A |
| GSE85916geoMatrix_GSM2287375 | B |
| GSE85916geoMatrix_GSM2287376 | A |
| GSE85916geoMatrix_GSM2287377 | A |
| GSE85916geoMatrix_GSM2287378 | A |
| GSE85916geoMatrix_GSM2287379 | A |
| GSE85916geoMatrix_GSM2287380 | A |
| GSE85916geoMatrix_GSM2287381 | B |
| GSE85916geoMatrix_GSM2287382 | B |
| GSE85916geoMatrix_GSM2287383 | A |

**Supplementary Table 3.** GO analysis of differentially expressed genes between two m^6^A modified subtypes.

| ONTOLOGY | ID | Description | pvalue | qvalue |
| --- | --- | --- | --- | --- |
| BP | GO:0006735 | NADH regeneration | 1.32E-07 | 0.000208428 |
| BP | GO:0061621 | canonical glycolysis | 1.32E-07 | 0.000208428 |
| BP | GO:0061718 | glucose catabolic process  to pyruvate | 1.32E-07 | 0.000208428 |
| BP | GO:0061620 | glycolytic process  through glucose-6-phosphate | 2.06E-07 | 0.00024398 |
| BP | GO:0061615 | glycolytic process  through fructose-6-phosphate | 3.14E-07 | 0.000297993 |
| BP | GO:0006007 | glucose catabolic process | 4.70E-07 | 0.000315514 |
| BP | GO:0019674 | NAD metabolic process | 5.94E-07 | 0.000315514 |
| BP | GO:1990868 | response to chemokine | 5.99E-07 | 0.000315514 |
| BP | GO:1990869 | cellular response to chemokine | 5.99E-07 | 0.000315514 |
| BP | GO:0097191 | extrinsic apoptotic  signaling pathway | 1.40E-06 | 0.000661729 |
| BP | GO:0045785 | positive regulation  of cell adhesion | 1.94E-06 | 0.000780618 |
| BP | GO:0032496 | response to lipopolysaccharide | 1.98E-06 | 0.000780618 |
| BP | GO:0042326 | negative regulation  of phosphorylation | 3.11E-06 | 0.001133422 |
| BP | GO:0007044 | cell-substrate junction assembly | 3.54E-06 | 0.001196778 |
| BP | GO:0150115 | cell-substrate junction organization | 5.60E-06 | 0.001768991 |
| BP | GO:1900739 | regulation of protein insertion  into mitochondrial membrane  involved in apoptotic signaling pathway | 9.20E-06 | 0.002563297 |
| BP | GO:1900740 | positive regulation of protein insertion into  mitochondrialmembrane involved in  apoptotic signaling pathway | 9.20E-06 | 0.002563297 |
| BP | GO:0002237 | response to molecule of  bacterial origin | 9.95E-06 | 0.002594334 |
| BP | GO:0070098 | chemokine-mediated signaling pathway | 1.04E-05 | 0.002594334 |
| BP | GO:0001933 | negative regulation of  protein phosphorylation | 1.19E-05 | 0.00280941 |
| BP | GO:0001704 | formation of primary germ layer | 1.28E-05 | 0.002896667 |
| BP | GO:0019320 | hexose catabolic process | 1.44E-05 | 0.003105304 |
| BP | GO:0006734 | NADH metabolic process | 1.51E-05 | 0.003107627 |
| BP | GO:0043405 | regulation of MAP kinase activity | 1.97E-05 | 0.003687684 |
| BP | GO:1901030 | positive regulation of mitochondrial  outer membrane permeabilization involved  in apoptotic signaling pathway | 2.01E-05 | 0.003687684 |
| BP | GO:0061041 | regulation of wound healing | 2.02E-05 | 0.003687684 |
| BP | GO:0050808 | synapse organization | 2.72E-05 | 0.004768874 |
| BP | GO:0071222 | cellular response to lipopolysaccharide | 2.86E-05 | 0.004836442 |
| BP | GO:0001844 | protein insertion into mitochondrial  membrane involved in apoptotic  signaling pathway | 3.41E-05 | 0.005573352 |
| BP | GO:0007369 | gastrulation | 3.74E-05 | 0.005750639 |
| BP | GO:0007159 | leukocyte cell-cell adhesion | 3.76E-05 | 0.005750639 |
| BP | GO:0001894 | tissue homeostasis | 5.24E-05 | 0.007764466 |
| BP | GO:0072655 | establishment of protein localization  to mitochondrion | 5.75E-05 | 0.007779187 |
| BP | GO:1903034 | regulation of response to wounding | 5.89E-05 | 0.007779187 |
| BP | GO:0034329 | cell junction assembly | 5.91E-05 | 0.007779187 |
| BP | GO:0052548 | regulation of endopeptidase activity | 5.91E-05 | 0.007779187 |
| BP | GO:0090183 | regulation of kidney development | 7.13E-05 | 0.008895158 |
| BP | GO:0090184 | positive regulation of  kidney development | 7.13E-05 | 0.008895158 |
| BP | GO:0022407 | regulation of cell-cell adhesion | 7.78E-05 | 0.009456269 |
| BP | GO:1903035 | negative regulation of  response to wounding | 8.25E-05 | 0.009738412 |
| BP | GO:1990778 | protein localization  to cell periphery | 8.43E-05 | 0.009738412 |
| BP | GO:0070585 | protein localization  to mitochondrion | 8.73E-05 | 0.009850555 |
| BP | GO:0046365 | monosaccharide catabolic process | 9.09E-05 | 0.009916464 |
| BP | GO:0071219 | cellular response to  molecule of bacterial origin | 9.21E-05 | 0.009916464 |
| BP | GO:0072659 | protein localization to  plasma membrane | 9.43E-05 | 0.009924056 |
| BP | GO:1903749 | positive regulation of  establishment of protein  localization to mitochondrion | 0.000102342 | 0.010541019 |
| BP | GO:0050863 | regulation of T cell activation | 0.000110213 | 0.010889733 |
| BP | GO:0050671 | positive regulation of  lymphocyte proliferation | 0.000113324 | 0.010889733 |
| BP | GO:0071902 | positive regulation of  protein serine/threonine kinase activity | 0.00011457 | 0.010889733 |
| BP | GO:0045862 | positive regulation of proteolysis | 0.000114922 | 0.010889733 |
| BP | GO:0050870 | positive regulation of  T cell activation | 0.000121278 | 0.011012679 |
| BP | GO:0033630 | positive regulation of  cell adhesion mediated by integrin | 0.000123192 | 0.011012679 |
| BP | GO:0046641 | positive regulation of  alpha-beta T cell proliferation | 0.000123192 | 0.011012679 |
| BP | GO:0032946 | positive regulation of  mononuclear cell proliferation | 0.000125595 | 0.011019558 |
| BP | GO:0061045 | negative regulation of  wound healing | 0.000141716 | 0.01220793 |
| BP | GO:2001233 | regulation of apoptotic  signaling pathway | 0.000151778 | 0.01259324 |
| BP | GO:2001235 | positive regulation of  apoptotic signaling pathway | 0.000156216 | 0.01259324 |
| BP | GO:0006835 | dicarboxylic acid transport | 0.000156433 | 0.01259324 |
| BP | GO:0048762 | mesenchymal cell differentiation | 0.000157519 | 0.01259324 |
| BP | GO:0052547 | regulation of peptidase activity | 0.000162437 | 0.01259324 |
| BP | GO:1905332 | positive regulation of morphogenesis  of an epithelium | 0.000163814 | 0.01259324 |
| BP | GO:0050804 | modulation of chemical  synaptic transmission | 0.000170973 | 0.01259324 |
| BP | GO:0090189 | regulation of branching involved  in ureteric bud morphogenesis | 0.00017167 | 0.01259324 |
| BP | GO:2001236 | regulation of extrinsic apoptotic  signaling pathway | 0.000174332 | 0.01259324 |
| BP | GO:0055074 | calcium ion homeostasis | 0.000179451 | 0.01259324 |
| BP | GO:0099177 | regulation of trans-synaptic signaling | 0.00017982 | 0.01259324 |
| BP | GO:0042035 | regulation of cytokine  biosynthetic process | 0.000182372 | 0.01259324 |
| BP | GO:1901224 | positive regulation of  NIK/NF-kappaB signaling | 0.000187387 | 0.01259324 |
| BP | GO:0060249 | anatomical structure homeostasis | 0.000188548 | 0.01259324 |
| BP | GO:0010469 | regulation of signaling  receptor activity | 0.000188717 | 0.01259324 |
| BP | GO:0035725 | sodium ion transmembrane transport | 0.000188717 | 0.01259324 |
| BP | GO:0072009 | nephron epithelium development | 0.000196572 | 0.012741699 |
| BP | GO:0015800 | acidic amino acid transport | 0.000197402 | 0.012741699 |
| BP | GO:0051098 | regulation of binding | 0.000199009 | 0.012741699 |
| BP | GO:1901028 | regulation of mitochondrial outer  membrane permeabilization involved  in apoptotic signaling pathway | 0.000206106 | 0.013020141 |
| BP | GO:0001656 | metanephros development | 0.000223029 | 0.01379506 |
| BP | GO:0042102 | positive regulation of  T cell proliferation | 0.000224197 | 0.01379506 |
| BP | GO:0071216 | cellular response to  biotic stimulus | 0.000228743 | 0.013886631 |
| BP | GO:0034110 | regulation of homotypic  cell-cell adhesion | 0.000233269 | 0.013886631 |
| BP | GO:0072170 | metanephric tubule development | 0.000234478 | 0.013886631 |
| BP | GO:0000302 | response to reactive oxygen species | 0.000243758 | 0.013953772 |
| BP | GO:1903039 | positive regulation of  leukocyte cell-cell adhesion | 0.000243758 | 0.013953772 |
| BP | GO:0006165 | nucleoside diphosphate phosphorylation | 0.000244447 | 0.013953772 |
| BP | GO:0032570 | response to progesterone | 0.000249813 | 0.014090341 |
| BP | GO:1903747 | regulation of establishment of  protein localization to mitochondrion | 0.000263214 | 0.014671536 |
| BP | GO:0001706 | endoderm formation | 0.000270784 | 0.014917977 |
| BP | GO:0072080 | nephron tubule development | 0.000285091 | 0.015525661 |
| BP | GO:0051101 | regulation of DNA binding | 0.000288368 | 0.015525661 |
| BP | GO:0046939 | nucleotide phosphorylation | 0.000297087 | 0.015815351 |
| BP | GO:0042542 | response to hydrogen peroxide | 0.000300489 | 0.015818733 |
| BP | GO:0032506 | cytokinetic process | 0.00031515 | 0.016277216 |
| BP | GO:0007200 | phospholipase C-activating  G protein-coupled receptor  signaling pathway | 0.000316069 | 0.016277216 |
| BP | GO:0060993 | kidney morphogenesis | 0.000321341 | 0.016370735 |
| BP | GO:1903829 | positive regulation of  cellular protein localization | 0.000329603 | 0.016613019 |
| BP | GO:0006096 | glycolytic process | 0.000338252 | 0.016812884 |
| BP | GO:0043406 | positive regulation of  MAP kinase activity | 0.000340665 | 0.016812884 |
| BP | GO:0001707 | mesoderm formation | 0.000346915 | 0.016944817 |
| BP | GO:0061326 | renal tubule development | 0.000361483 | 0.017425641 |
| BP | GO:0031581 | hemidesmosome assembly | 0.000366659 | 0.017425641 |
| BP | GO:0042110 | T cell activation | 0.000367793 | 0.017425641 |
| BP | GO:0006757 | ATP generation from ADP | 0.000375324 | 0.017606405 |
| BP | GO:0090190 | positive regulation of branching  involved in ureteric bud morphogenesis | 0.000382414 | 0.017763115 |
| BP | GO:1903037 | regulation of leukocyte cell-cell adhesion | 0.000405007 | 0.018406638 |
| BP | GO:0022409 | positive regulation of cell-cell adhesion | 0.000405021 | 0.018406638 |
| BP | GO:0034109 | homotypic cell-cell adhesion | 0.000407923 | 0.018406638 |
| BP | GO:0061217 | regulation of mesonephros development | 0.000415141 | 0.018555589 |
| BP | GO:0001558 | regulation of cell growth | 0.000423581 | 0.018602204 |
| BP | GO:0002791 | regulation of peptide secretion | 0.000424036 | 0.018602204 |
| BP | GO:0070665 | positive regulation of  leukocyte proliferation | 0.000430365 | 0.018706636 |
| BP | GO:0001819 | positive regulation of  cytokine production | 0.000438241 | 0.018875814 |
| BP | GO:0048332 | mesoderm morphogenesis | 0.00045226 | 0.019304144 |
| BP | GO:0051480 | regulation of cytosolic  calcium ion concentration | 0.000465065 | 0.019520627 |
| BP | GO:1904951 | positive regulation of  establishment of protein localization | 0.000468402 | 0.019520627 |
| BP | GO:0044262 | cellular carbohydrate metabolic process | 0.000473702 | 0.019520627 |
| BP | GO:0046640 | regulation of alpha-beta  T cell proliferation | 0.000473812 | 0.019520627 |
| BP | GO:0006090 | pyruvate metabolic process | 0.000511909 | 0.02073068 |
| BP | GO:0001658 | branching involved in  ureteric bud morphogenesis | 0.000512184 | 0.02073068 |
| BP | GO:0050818 | regulation of coagulation | 0.000519068 | 0.02073068 |
| BP | GO:0031349 | positive regulation of  defense response | 0.000520685 | 0.02073068 |
| BP | GO:0072077 | renal vesicle morphogenesis | 0.000531616 | 0.020989494 |
| BP | GO:0072202 | cell differentiation involved in  metanephros development | 0.000539858 | 0.021138755 |
| BP | GO:0007204 | positive regulation of  cytosolic calcium ion concentration | 0.000556739 | 0.021168216 |
| BP | GO:0002687 | positive regulation of  leukocyte migration | 0.000566842 | 0.021168216 |
| BP | GO:0072073 | kidney epithelium development | 0.000566842 | 0.021168216 |
| BP | GO:0071470 | cellular response to osmotic stress | 0.000566918 | 0.021168216 |
| BP | GO:0072182 | regulation of nephron tubule  epithelial cell differentiation | 0.000567417 | 0.021168216 |
| BP | GO:0098712 | glutamate import across  plasma membrane | 0.000567417 | 0.021168216 |
| BP | GO:0007492 | endoderm development | 0.000583543 | 0.021599721 |
| BP | GO:0016052 | carbohydrate catabolic process | 0.000592145 | 0.021748215 |
| BP | GO:0072006 | nephron development | 0.000618918 | 0.022556682 |
| BP | GO:0001763 | morphogenesis of  a branching structure | 0.000636346 | 0.023014814 |
| BP | GO:0009132 | nucleoside diphosphate  metabolic process | 0.000659264 | 0.023485133 |
| BP | GO:0048754 | branching morphogenesis of  an epithelial tube | 0.000659264 | 0.023485133 |
| BP | GO:0006874 | cellular calcium ion homeostasis | 0.000665337 | 0.023524613 |
| BP | GO:0070661 | leukocyte proliferation | 0.000684108 | 0.023592849 |
| BP | GO:0042129 | regulation of T cell proliferation | 0.000690116 | 0.023592849 |
| BP | GO:0072207 | metanephric epithelium development | 0.000692627 | 0.023592849 |
| BP | GO:0050708 | regulation of protein secretion | 0.000704254 | 0.023592849 |
| BP | GO:0060425 | lung morphogenesis | 0.000706721 | 0.023592849 |
| BP | GO:0042089 | cytokine biosynthetic process | 0.000722404 | 0.023592849 |
| BP | GO:0060231 | mesenchymal to epithelial transition | 0.000722404 | 0.023592849 |
| BP | GO:0072087 | renal vesicle development | 0.000722404 | 0.023592849 |
| BP | GO:0048333 | mesodermal cell differentiation | 0.000724396 | 0.023592849 |
| BP | GO:0110111 | negative regulation of  animal organ morphogenesis | 0.000724396 | 0.023592849 |
| BP | GO:0033627 | cell adhesion mediated by integrin | 0.000733386 | 0.023592849 |
| BP | GO:0051899 | membrane depolarization | 0.000733494 | 0.023592849 |
| BP | GO:0038127 | ERBB signaling pathway | 0.000735555 | 0.023592849 |
| BP | GO:0033157 | regulation of intracellular  protein transport | 0.000736982 | 0.023592849 |
| BP | GO:0098739 | import across plasma membrane | 0.000776712 | 0.02423039 |
| BP | GO:0060485 | mesenchyme development | 0.000779908 | 0.02423039 |
| BP | GO:0051222 | positive regulation of  protein transport | 0.000780857 | 0.02423039 |
| BP | GO:0051346 | negative regulation of  hydrolase activity | 0.000781173 | 0.02423039 |
| BP | GO:0022600 | digestive system process | 0.000782468 | 0.02423039 |
| BP | GO:0060393 | regulation of pathway-restricted  SMAD protein phosphorylation | 0.000793447 | 0.024410823 |
| BP | GO:0045165 | cell fate commitment | 0.000806526 | 0.024585447 |
| BP | GO:0006814 | sodium ion transport | 0.000819491 | 0.024585447 |
| BP | GO:0046031 | ADP metabolic process | 0.000820339 | 0.024585447 |
| BP | GO:0048168 | regulation of neuronal  synaptic plasticity | 0.000826457 | 0.024585447 |
| BP | GO:0010950 | positive regulation of  endopeptidase activity | 0.000827477 | 0.024585447 |
| BP | GO:0000281 | mitotic cytokinesis | 0.000834406 | 0.024585447 |
| BP | GO:0030193 | regulation of blood coagulation | 0.000839912 | 0.024585447 |
| BP | GO:0051938 | L-glutamate import | 0.000840635 | 0.024585447 |
| BP | GO:0034765 | regulation of ion  transmembrane transport | 0.000870624 | 0.02496919 |
| BP | GO:0046633 | alpha-beta T cell proliferation | 0.000884562 | 0.02496919 |
| BP | GO:0009135 | purine nucleoside diphosphate  metabolic process | 0.000890709 | 0.02496919 |
| BP | GO:0009179 | purine ribonucleoside  diphosphate metabolic process | 0.000890709 | 0.02496919 |
| BP | GO:0090316 | positive regulation of  intracellular protein transport | 0.000890936 | 0.02496919 |
| BP | GO:0032147 | activation of protein  kinase activity | 0.00090585 | 0.02496919 |
| BP | GO:0002283 | neutrophil activation involved  in immune response | 0.000908467 | 0.02496919 |
| BP | GO:0046635 | positive regulation of alpha-beta  T cell activation | 0.000911728 | 0.02496919 |
| BP | GO:0051893 | regulation of focal adhesion assembly | 0.000911728 | 0.02496919 |
| BP | GO:0070059 | intrinsic apoptotic signaling pathway  in response to endoplasmic  reticulum stress | 0.000911728 | 0.02496919 |
| BP | GO:0090109 | regulation of cell-substrate  junction assembly | 0.000911728 | 0.02496919 |
| BP | GO:0048041 | focal adhesion assembly | 0.000943993 | 0.025031997 |
| BP | GO:1900046 | regulation of hemostasis | 0.000943993 | 0.025031997 |
| BP | GO:0048738 | cardiac muscle tissue development | 0.000946837 | 0.025031997 |
| BP | GO:0050670 | regulation of lymphocyte proliferation | 0.000959802 | 0.025031997 |
| BP | GO:0042107 | cytokine metabolic process | 0.000962088 | 0.025031997 |
| BP | GO:0061213 | positive regulation of  mesonephros development | 0.000962088 | 0.025031997 |
| BP | GO:0014047 | glutamate secretion | 0.00096357 | 0.025031997 |
| BP | GO:0045214 | sarcomere organization | 0.00096357 | 0.025031997 |
| BP | GO:0051204 | protein insertion into  mitochondrial membrane | 0.00096357 | 0.025031997 |
| BP | GO:0042098 | T cell proliferation | 0.000966855 | 0.025031997 |
| BP | GO:0031623 | receptor internalization | 0.000982494 | 0.025298646 |
| BP | GO:0072503 | cellular divalent inorganic  cation homeostasis | 0.000988546 | 0.02531689 |
| BP | GO:0007565 | female pregnancy | 0.001036767 | 0.026409102 |
| BP | GO:2001234 | negative regulation of  apoptotic signaling pathway | 0.001074726 | 0.027229612 |
| BP | GO:0032944 | regulation of mononuclear  cell proliferation | 0.00109342 | 0.027555896 |
| BP | GO:0070979 | protein K11-linked ubiquitination | 0.001099646 | 0.027565577 |
| BP | GO:0050819 | negative regulation of coagulation | 0.001116052 | 0.027565577 |
| BP | GO:0097345 | mitochondrial outer membrane  permeabilization | 0.001116052 | 0.027565577 |
| BP | GO:0006469 | negative regulation of  protein kinase activity | 0.001117076 | 0.027565577 |
| BP | GO:0009185 | ribonucleoside diphosphate  metabolic process | 0.001150529 | 0.028169949 |
| BP | GO:0032388 | positive regulation of  intracellular transport | 0.001166059 | 0.028169949 |
| BP | GO:0009914 | hormone transport | 0.001170982 | 0.028169949 |
| BP | GO:0006970 | response to osmotic stress | 0.001184725 | 0.028169949 |
| BP | GO:0035418 | protein localization to synapse | 0.001184725 | 0.028169949 |
| BP | GO:0032729 | positive regulation of  interferon-gamma production | 0.001192196 | 0.028169949 |
| BP | GO:0060389 | pathway-restricted SMAD  protein phosphorylation | 0.001192196 | 0.028169949 |
| BP | GO:0060675 | ureteric bud morphogenesis | 0.001192196 | 0.028169949 |
| BP | GO:0045651 | positive regulation of  macrophage differentiation | 0.001201025 | 0.028169949 |
| BP | GO:0072160 | nephron tubule epithelial  cell differentiation | 0.001201025 | 0.028169949 |
| BP | GO:0072088 | nephron epithelium morphogenesis | 0.001209455 | 0.028227937 |
| BP | GO:1903362 | regulation of cellular protein  catabolic process | 0.001221639 | 0.028372543 |
| BP | GO:0042391 | regulation of membrane potential | 0.001229997 | 0.028427306 |
| BP | GO:0061138 | morphogenesis of a  branching epithelium | 0.001275144 | 0.029327666 |
| BP | GO:0099175 | regulation of postsynapse  organization | 0.001291593 | 0.029420339 |
| BP | GO:2001237 | negative regulation of extrinsic  apoptotic signaling pathway | 0.001291593 | 0.029420339 |
| BP | GO:0001837 | epithelial to mesenchymal transition | 0.001304533 | 0.029572916 |
| BP | GO:0002685 | regulation of leukocyte migration | 0.001346844 | 0.030326824 |
| BP | GO:0072171 | mesonephric tubule morphogenesis | 0.001356992 | 0.030326824 |
| BP | GO:0150116 | regulation of cell-substrate  junction organization | 0.001356992 | 0.030326824 |
| BP | GO:0048704 | embryonic skeletal system morphogenesis | 0.001391077 | 0.030654785 |
| BP | GO:1990823 | response to leukemia inhibitory factor | 0.001391077 | 0.030654785 |
| BP | GO:1990830 | cellular response to leukemia  inhibitory factor | 0.001391077 | 0.030654785 |
| BP | GO:0043312 | neutrophil degranulation | 0.00141319 | 0.030997903 |
| BP | GO:0010822 | positive regulation of  mitochondrion organization | 0.001421207 | 0.031030091 |
| BP | GO:0046879 | hormone secretion | 0.001436572 | 0.031079355 |
| BP | GO:0016051 | carbohydrate biosynthetic process | 0.001436583 | 0.031079355 |
| BP | GO:0044706 | multi-multicellular organism process | 0.001499859 | 0.032300799 |
| BP | GO:0046902 | regulation of mitochondrial  membrane permeability | 0.001529787 | 0.032648507 |
| BP | GO:0072028 | nephron morphogenesis | 0.001529787 | 0.032648507 |
| BP | GO:0001657 | ureteric bud development | 0.001538134 | 0.032679452 |
| BP | GO:0097193 | intrinsic apoptotic  signaling pathway | 0.001578934 | 0.033396523 |
| BP | GO:1902410 | mitotic cytokinetic process | 0.001619913 | 0.034111011 |
| BP | GO:0008544 | epidermis development | 0.001641444 | 0.034381911 |
| BP | GO:0070663 | regulation of leukocyte proliferation | 0.001647292 | 0.034381911 |
| BP | GO:0072283 | metanephric renal  vesicle morphogenesis | 0.001664049 | 0.03457934 |
| BP | GO:0048705 | skeletal system morphogenesis | 0.001695529 | 0.034824886 |
| BP | GO:0072163 | mesonephric epithelium development | 0.001697916 | 0.034824886 |
| BP | GO:0072164 | mesonephric tubule development | 0.001697916 | 0.034824886 |
| BP | GO:0060562 | epithelial tube morphogenesis | 0.001755725 | 0.035855349 |
| BP | GO:0000187 | activation of MAPK activity | 0.001773119 | 0.035968564 |
| BP | GO:1903897 | regulation of PERK-mediated  unfolded protein response | 0.001786862 | 0.035968564 |
| BP | GO:0019318 | hexose metabolic process | 0.001787992 | 0.035968564 |
| BP | GO:0032386 | regulation of intracellular transport | 0.001811127 | 0.035968564 |
| BP | GO:0003254 | regulation of membrane depolarization | 0.001814411 | 0.035968564 |
| BP | GO:0090151 | establishment of protein localization  to mitochondrial membrane | 0.001814411 | 0.035968564 |
| BP | GO:2000107 | negative regulation of leukocyte  apoptotic process | 0.001814411 | 0.035968564 |
| BP | GO:0032892 | positive regulation of  organic acid transport | 0.001829913 | 0.036124721 |
| BP | GO:0051604 | protein maturation | 0.001851027 | 0.036389921 |
| BP | GO:0050673 | epithelial cell proliferation | 0.001880255 | 0.036811787 |
| BP | GO:0070373 | negative regulation of  ERK1 and ERK2 cascade | 0.001916951 | 0.037303639 |
| BP | GO:0033673 | negative regulation of  kinase activity | 0.001921125 | 0.037303639 |
| BP | GO:0007498 | mesoderm development | 0.001949363 | 0.037697454 |
| BP | GO:0062197 | cellular response to  chemical stress | 0.001988818 | 0.038304102 |
| BP | GO:0005976 | polysaccharide metabolic process | 0.002054017 | 0.039399669 |
| BP | GO:0051051 | negative regulation of transport | 0.002088176 | 0.039813163 |
| BP | GO:0010718 | positive regulation of epithelial  to mesenchymal transition | 0.002100783 | 0.039813163 |
| BP | GO:0030195 | negative regulation of  blood coagulation | 0.002100783 | 0.039813163 |
| BP | GO:0071692 | protein localization to  extracellular region | 0.00215136 | 0.04033926 |
| BP | GO:0001822 | kidney development | 0.002157026 | 0.04033926 |
| BP | GO:0046651 | lymphocyte proliferation | 0.002157026 | 0.04033926 |
| BP | GO:0005996 | monosaccharide metabolic process | 0.0021626 | 0.04033926 |
| BP | GO:1902107 | positive regulation of  leukocyte differentiation | 0.002211682 | 0.040766145 |
| BP | GO:0030856 | regulation of epithelial  cell differentiation | 0.002221658 | 0.040766145 |
| BP | GO:0034103 | regulation of tissue remodeling | 0.002231795 | 0.040766145 |
| BP | GO:0030033 | microvillus assembly | 0.002245716 | 0.040766145 |
| BP | GO:0061952 | midbody abscission | 0.002245716 | 0.040766145 |
| BP | GO:1901673 | regulation of mitotic spindle assembly | 0.002245716 | 0.040766145 |
| BP | GO:2000811 | negative regulation of anoikis | 0.002245716 | 0.040766145 |
| BP | GO:0007411 | axon guidance | 0.002273055 | 0.041104948 |
| BP | GO:0008637 | apoptotic mitochondrial changes | 0.002380795 | 0.04288956 |
| BP | GO:0097485 | neuron projection guidance | 0.002394391 | 0.042971101 |
| BP | GO:0030900 | forebrain development | 0.002405619 | 0.043009699 |
| BP | GO:1900047 | negative regulation of hemostasis | 0.002422027 | 0.043140251 |
| BP | GO:0003338 | metanephros morphogenesis | 0.002457525 | 0.043284374 |
| BP | GO:0006536 | glutamate metabolic process | 0.002457525 | 0.043284374 |
| BP | GO:0043552 | positive regulation of  phosphatidylinositol 3-kinase activity | 0.002457525 | 0.043284374 |
| BP | GO:0001823 | mesonephros development | 0.002481453 | 0.043470233 |
| BP | GO:0070371 | ERK1 and ERK2 cascade | 0.002486428 | 0.043470233 |
| BP | GO:0097529 | myeloid leukocyte migration | 0.002502472 | 0.043508668 |
| BP | GO:1902110 | positive regulation of mitochondrial  membrane permeability involved in  apoptotic process | 0.002512729 | 0.043508668 |
| BP | GO:0032943 | mononuclear cell proliferation | 0.002521226 | 0.043508668 |
| BP | GO:1903364 | positive regulation of cellular  protein catabolic process | 0.002528813 | 0.043508668 |
| BP | GO:0001709 | cell fate determination | 0.002534542 | 0.043508668 |
| BP | GO:0099173 | postsynapse organization | 0.002548843 | 0.0435962 |
| BP | GO:0015740 | C4-dicarboxylate transport | 0.002572897 | 0.043622275 |
| BP | GO:0032528 | microvillus organization | 0.002572897 | 0.043622275 |
| BP | GO:0002573 | myeloid leukocyte differentiation | 0.002577988 | 0.043622275 |
| BP | GO:0048703 | embryonic viscerocranium morphogenesis | 0.002679578 | 0.045019714 |
| BP | GO:1905383 | protein localization to presynapse | 0.002679578 | 0.045019714 |
| BP | GO:0009306 | protein secretion | 0.002703298 | 0.04525774 |
| BP | GO:0120034 | positive regulation of plasma membrane  bounded cell projection assembly | 0.002718017 | 0.045335114 |
| BP | GO:0051099 | positive regulation of binding | 0.002727057 | 0.045335114 |
| BP | GO:0016049 | cell growth | 0.002739866 | 0.045388804 |
| BP | GO:0035592 | establishment of protein localization  to extracellular region | 0.002811571 | 0.046414389 |
| BP | GO:0034113 | heterotypic cell-cell adhesion | 0.002843361 | 0.046776195 |
| BP | GO:0043409 | negative regulation of MAPK cascade | 0.002873283 | 0.04710489 |
| BP | GO:2001056 | positive regulation of cysteine-type  endopeptidase activity | 0.002927129 | 0.047737697 |
| BP | GO:0019228 | neuronal action potential | 0.00294211 | 0.047737697 |
| BP | GO:0051482 | positive regulation of cytosolic  calcium ion concentration involved  in phospholipase C-activating G  protein-coupled signaling pathway | 0.00294211 | 0.047737697 |
| BP | GO:0032740 | positive regulation of  interleukin-17 production | 0.00296237 | 0.047739446 |
| BP | GO:0035994 | response to muscle stretch | 0.00296237 | 0.047739446 |
| BP | GO:1902414 | protein localization to cell junction | 0.002972799 | 0.047745114 |
| BP | GO:0001701 | in utero embryonic development | 0.00305325 | 0.048654932 |
| BP | GO:0043123 | positive regulation of I-kappaB  kinase/NF-kappaB signaling | 0.003062692 | 0.048654932 |
| BP | GO:0051783 | regulation of nuclear division | 0.003062692 | 0.048654932 |
| BP | GO:0002833 | positive regulation of response  to biotic stimulus | 0.003070525 | 0.048654932 |
| BP | GO:0072078 | nephron tubule morphogenesis | 0.003108265 | 0.049088771 |
| BP | GO:0044282 | small molecule catabolic process | 0.003137055 | 0.049345171 |
| BP | GO:0043392 | negative regulation of DNA binding | 0.003180846 | 0.049345171 |
| BP | GO:0010952 | positive regulation of  peptidase activity | 0.003181347 | 0.049345171 |
| BP | GO:0051156 | glucose 6-phosphate metabolic process | 0.003182808 | 0.049345171 |
| BP | GO:0072273 | metanephric nephron morphogenesis | 0.003182808 | 0.049345171 |
| BP | GO:0006081 | cellular aldehyde metabolic process | 0.00320782 | 0.049345171 |
| BP | GO:0055008 | cardiac muscle tissue morphogenesis | 0.00320782 | 0.049345171 |
| BP | GO:1902686 | mitochondrial outer membrane  permeabilization involved in  programmed cell death | 0.00320782 | 0.049345171 |
| BP | GO:0036293 | response to decreased oxygen levels | 0.003260198 | 0.04998859 |
| CC | GO:0098978 | glutamatergic synapse | 2.30E-06 | 0.001239022 |
| CC | GO:0005911 | cell-cell junction | 4.63E-06 | 0.001248923 |
| CC | GO:0034703 | cation channel complex | 1.15E-05 | 0.002069221 |
| CC | GO:0005925 | focal adhesion | 1.71E-05 | 0.002310003 |
| CC | GO:0030055 | cell-substrate junction | 2.78E-05 | 0.002991753 |
| CC | GO:0031674 | I band | 5.33E-05 | 0.004345056 |
| CC | GO:0034702 | ion channel complex | 5.64E-05 | 0.004345056 |
| CC | GO:0070820 | tertiary granule | 7.04E-05 | 0.004743511 |
| CC | GO:0005938 | cell cortex | 8.45E-05 | 0.005061497 |
| CC | GO:0009925 | basal plasma membrane | 0.000127142 | 0.006226083 |
| CC | GO:0030018 | Z disc | 0.000137097 | 0.006226083 |
| CC | GO:0045178 | basal part of cell | 0.000138628 | 0.006226083 |
| CC | GO:1990351 | transporter complex | 0.00018734 | 0.007766641 |
| CC | GO:1902495 | transmembrane transporter complex | 0.000245075 | 0.009434448 |
| CC | GO:0001726 | ruffle | 0.000266312 | 0.009568553 |
| CC | GO:0005903 | brush border | 0.000371382 | 0.012509703 |
| CC | GO:0097386 | glial cell projection | 0.000567632 | 0.017995524 |
| CC | GO:0030016 | myofibril | 0.000675465 | 0.020224446 |
| CC | GO:0030133 | transport vesicle | 0.000840006 | 0.023827321 |
| CC | GO:0030017 | sarcomere | 0.000898805 | 0.024220432 |
| CC | GO:1904724 | tertiary granule lumen | 0.001071161 | 0.027490459 |
| CC | GO:0033268 | node of Ranvier | 0.001171898 | 0.028708702 |
| CC | GO:0043292 | contractile fiber | 0.001356476 | 0.03178561 |
| CC | GO:0001772 | immunological synapse | 0.001488728 | 0.033431093 |
| CC | GO:0016528 | sarcoplasm | 0.002044768 | 0.043170171 |
| CC | GO:0098862 | cluster of actin-based cell projections | 0.002082624 | 0.043170171 |
| CC | GO:0001518 | voltage-gated sodium channel complex | 0.002192404 | 0.04376261 |
| CC | GO:0099738 | cell cortex region | 0.002450383 | 0.047165269 |
| MF | GO:0045296 | cadherin binding | 6.76E-07 | 0.000601778 |
| MF | GO:0005126 | cytokine receptor binding | 2.54E-06 | 0.001130048 |
| MF | GO:0005125 | cytokine activity | 4.75E-06 | 0.00140871 |
| MF | GO:0044389 | ubiquitin-like protein ligase binding | 1.58E-05 | 0.003514042 |
| MF | GO:0048018 | receptor ligand activity | 4.09E-05 | 0.006819046 |
| MF | GO:0098918 | structural constituent of synapse | 4.92E-05 | 0.006819046 |
| MF | GO:0030546 | signaling receptor activator activity | 5.36E-05 | 0.006819046 |
| MF | GO:0031625 | ubiquitin protein ligase binding | 0.000108543 | 0.012082534 |
| MF | GO:0000774 | adenyl-nucleotide exchange factor activity | 0.000151845 | 0.015024671 |
| MF | GO:0042379 | chemokine receptor binding | 0.000239575 | 0.02104237 |
| MF | GO:0051400 | BH domain binding | 0.000264602 | 0.02104237 |
| MF | GO:0008083 | growth factor activity | 0.00028355 | 0.02104237 |
| MF | GO:0045236 | CXCR chemokine receptor binding | 0.000461316 | 0.031601052 |
| MF | GO:0043621 | protein self-association | 0.000504308 | 0.032078547 |
| MF | GO:0003779 | actin binding | 0.000559822 | 0.033235732 |
| MF | GO:0098631 | cell adhesion mediator activity | 0.000794351 | 0.044211892 |
| MF | GO:0005172 | vascular endothelial growth  factor receptor binding | 0.000985102 | 0.048736634 |
| MF | GO:0044548 | S100 protein binding | 0.000985102 | 0.048736634 |

**Supplementary Table 4.** KEGG analysis of differentially expressed genes between two m^6^A modified subtypes.

| ID | Description | pvalue | qvalue |
| --- | --- | --- | --- |
| hsa00010 | Glycolysis / Gluconeogenesis | 1.89E-05 | 0.005046635 |
| hsa05410 | Hypertrophic cardiomyopathy | 5.60E-05 | 0.005866933 |
| hsa04066 | HIF-1 signaling pathway | 6.61E-05 | 0.005866933 |
| hsa04215 | Apoptosis - multiple species | 0.000140865 | 0.009378635 |
| hsa05414 | Dilated cardiomyopathy | 0.000416665 | 0.021831864 |
| hsa05230 | Central carbon metabolism in cancer | 0.000491864 | 0.021831864 |
| hsa04621 | NOD-like receptor signaling pathway | 0.000632369 | 0.024058566 |
| hsa04060 | Cytokine-cytokine receptor interaction | 0.00074318 | 0.024740073 |
| hsa05412 | Arrhythmogenic right ventricular cardiomyopathy | 0.001253878 | 0.037103052 |
| hsa05418 | Fluid shear stress and atherosclerosis | 0.001576718 | 0.038905902 |
| hsa05205 | Proteoglycans in cancer | 0.001669036 | 0.038905902 |
| hsa00500 | Starch and sucrose metabolism | 0.001808785 | 0.038905902 |
| hsa04061 | Viral protein interaction with cytokine and cytokine receptor | 0.001899162 | 0.038905902 |
| hsa05222 | Small cell lung cancer | 0.002316272 | 0.042070601 |
| hsa05131 | Shigellosis | 0.002560742 | 0.042070601 |
| hsa04510 | Focal adhesion | 0.002719535 | 0.042070601 |
| hsa04390 | Hippo signaling pathway | 0.002828616 | 0.042070601 |
| hsa04350 | TGF-beta signaling pathway | 0.002843507 | 0.042070601 |
| hsa04610 | Complement and coagulation cascades | 0.003132628 | 0.043908863 |

**Supplementary Table 5.** Identification of m6A-related gene subtypes.

| ID | geneCluster |
| --- | --- |
| TCGA_TCGA-FB-AAQ1 | A |
| TCGA_TCGA-IB-7888 | B |
| TCGA_TCGA-2J-AABO | A |
| TCGA_TCGA-FB-A5VM | A |
| TCGA_TCGA-HZ-8315 | A |
| TCGA_TCGA-IB-7893 | A |
| TCGA_TCGA-2L-AAQE | A |
| TCGA_TCGA-HZ-8003 | B |
| TCGA_TCGA-YH-A8SY | A |
| TCGA_TCGA-M8-A5N4 | A |
| TCGA_TCGA-2J-AABT | A |
| TCGA_TCGA-IB-AAUU | A |
| TCGA_TCGA-PZ-A5RE | A |
| TCGA_TCGA-HV-A7OL | A |
| TCGA_TCGA-IB-7897 | B |
| TCGA_TCGA-H6-A45N | A |
| TCGA_TCGA-3A-A9IX | B |
| TCGA_TCGA-XD-AAUH | B |
| TCGA_TCGA-HZ-A77O | A |
| TCGA_TCGA-IB-A5SO | A |
| TCGA_TCGA-F2-6879 | A |
| TCGA_TCGA-3A-A9IV | B |
| TCGA_TCGA-HZ-8519 | B |
| TCGA_TCGA-3A-A9IR | B |
| TCGA_TCGA-HV-AA8V | A |
| TCGA_TCGA-Q3-A5QY | B |
| TCGA_TCGA-HZ-A9TJ | A |
| TCGA_TCGA-3A-A9IL | B |
| TCGA_TCGA-H6-8124 | A |
| TCGA_TCGA-3E-AAAY | A |
| TCGA_TCGA-LB-A8F3 | B |
| TCGA_TCGA-US-A77E | A |
| TCGA_TCGA-YY-A8LH | A |
| TCGA_TCGA-IB-7654 | A |
| TCGA_TCGA-FB-A78T | A |
| TCGA_TCGA-2J-AAB8 | A |
| TCGA_TCGA-FB-A4P6 | A |
| TCGA_TCGA-3A-A9I5 | B |
| TCGA_TCGA-HZ-8005 | A |
| TCGA_TCGA-2J-AAB9 | A |
| TCGA_TCGA-XN-A8T5 | A |
| TCGA_TCGA-HZ-8638 | A |
| TCGA_TCGA-L1-A7W4 | A |
| TCGA_TCGA-US-A779 | A |
| TCGA_TCGA-HZ-7925 | A |
| TCGA_TCGA-IB-7886 | A |
| TCGA_TCGA-IB-8127 | A |
| TCGA_TCGA-XN-A8T3 | A |
| TCGA_TCGA-FB-AAQ0 | A |
| TCGA_TCGA-S4-A8RM | A |
| TCGA_TCGA-IB-AAUP | A |
| TCGA_TCGA-HZ-8317 | A |
| TCGA_TCGA-3A-A9I9 | B |
| TCGA_TCGA-F2-6880 | B |
| TCGA_TCGA-2L-AAQJ | A |
| TCGA_TCGA-3A-A9IH | A |
| TCGA_TCGA-IB-A5ST | A |
| TCGA_TCGA-3A-A9IU | A |
| TCGA_TCGA-HV-AA8X | A |
| TCGA_TCGA-HZ-A77Q | A |
| TCGA_TCGA-HZ-7924 | A |
| TCGA_TCGA-HZ-7289 | A |
| TCGA_TCGA-HZ-7926 | A |
| TCGA_TCGA-RB-AA9M | A |
| TCGA_TCGA-3A-A9IO | B |
| TCGA_TCGA-2J-AABH | A |
| TCGA_TCGA-IB-7645 | A |
| TCGA_TCGA-2J-AABA | A |
| TCGA_TCGA-HZ-A4BH | A |
| TCGA_TCGA-Q3-AA2A | A |
| TCGA_TCGA-FB-AAQ2 | A |
| TCGA_TCGA-2L-AAQM | B |
| TCGA_TCGA-IB-7649 | B |
| TCGA_TCGA-LB-A9Q5 | B |
| TCGA_TCGA-HZ-A8P0 | A |
| TCGA_TCGA-S4-A8RO | A |
| TCGA_TCGA-3A-A9IC | A |
| TCGA_TCGA-IB-AAUN | A |
| TCGA_TCGA-HZ-A49G | A |
| TCGA_TCGA-S4-A8RP | A |
| TCGA_TCGA-HZ-A49H | B |
| TCGA_TCGA-IB-AAUM | B |
| TCGA_TCGA-HZ-A8P1 | A |
| TCGA_TCGA-2J-AABV | B |
| TCGA_TCGA-IB-A5SS | A |
| TCGA_TCGA-FB-AAPZ | A |
| TCGA_TCGA-2J-AABI | A |
| TCGA_TCGA-IB-AAUO | A |
| TCGA_TCGA-2J-AAB6 | A |
| TCGA_TCGA-2J-AABF | A |
| TCGA_TCGA-HZ-8002 | B |
| TCGA_TCGA-FB-AAQ6 | A |
| TCGA_TCGA-2J-AAB1 | A |
| TCGA_TCGA-HV-A5A6 | A |
| TCGA_TCGA-HZ-8001 | B |
| TCGA_TCGA-3E-AAAZ | A |
| TCGA_TCGA-IB-7885 | A |
| TCGA_TCGA-IB-7651 | A |
| TCGA_TCGA-HZ-7923 | B |
| TCGA_TCGA-HZ-8637 | B |
| TCGA_TCGA-HZ-A77P | B |
| TCGA_TCGA-IB-7646 | A |
| TCGA_TCGA-HV-A5A3 | A |
| TCGA_TCGA-HZ-7920 | B |
| TCGA_TCGA-FB-AAPU | A |
| TCGA_TCGA-HZ-7922 | A |
| TCGA_TCGA-IB-A6UF | A |
| TCGA_TCGA-OE-A75W | A |
| TCGA_TCGA-3A-A9IZ | A |
| TCGA_TCGA-3A-A9IN | B |
| TCGA_TCGA-F2-7276 | B |
| TCGA_TCGA-HV-A5A5 | B |
| TCGA_TCGA-2J-AABU | A |
| TCGA_TCGA-US-A77J | B |
| TCGA_TCGA-2J-AABE | A |
| TCGA_TCGA-RB-A7B8 | A |
| TCGA_TCGA-IB-A6UG | A |
| TCGA_TCGA-3A-A9J0 | A |
| TCGA_TCGA-IB-AAUQ | A |
| TCGA_TCGA-FB-A7DR | A |
| TCGA_TCGA-2L-AAQL | A |
| TCGA_TCGA-HZ-7918 | B |
| TCGA_TCGA-IB-7887 | A |
| TCGA_TCGA-2J-AABR | A |
| TCGA_TCGA-IB-A5SP | A |
| TCGA_TCGA-3A-A9I7 | A |
| TCGA_TCGA-FB-AAPP | B |
| TCGA_TCGA-F2-A7TX | A |
| TCGA_TCGA-XD-AAUL | A |
| TCGA_TCGA-F2-A44H | B |
| TCGA_TCGA-IB-7644 | A |
| TCGA_TCGA-3A-A9IB | A |
| TCGA_TCGA-IB-AAUW | B |
| TCGA_TCGA-HZ-7919 | A |
| TCGA_TCGA-F2-7273 | B |
| TCGA_TCGA-2L-AAQI | A |
| TCGA_TCGA-XD-AAUG | B |
| TCGA_TCGA-3A-A9IS | B |
| TCGA_TCGA-IB-AAUT | B |
| TCGA_TCGA-Z5-AAPL | A |
| TCGA_TCGA-HZ-A4BK | A |
| TCGA_TCGA-US-A776 | B |
| TCGA_TCGA-IB-7889 | A |
| TCGA_TCGA-IB-AAUR | A |
| TCGA_TCGA-IB-A7M4 | A |
| TCGA_TCGA-FB-AAPS | A |
| TCGA_TCGA-LB-A7SX | A |
| TCGA_TCGA-IB-AAUS | A |
| TCGA_TCGA-HV-A7OP | B |
| TCGA_TCGA-IB-7652 | A |
| TCGA_TCGA-2J-AAB4 | A |
| TCGA_TCGA-US-A774 | A |
| TCGA_TCGA-IB-8126 | B |
| TCGA_TCGA-F2-A44G | A |
| TCGA_TCGA-IB-AAUV | B |
| TCGA_TCGA-FB-AAQ3 | A |
| TCGA_TCGA-RL-AAAS | B |
| TCGA_TCGA-IB-7890 | A |
| TCGA_TCGA-XD-AAUI | A |
| TCGA_TCGA-2J-AABK | B |
| TCGA_TCGA-FB-A545 | A |
| TCGA_TCGA-3A-A9IJ | B |
| TCGA_TCGA-F2-A8YN | A |
| TCGA_TCGA-HZ-A49I | A |
| TCGA_TCGA-HV-A5A4 | A |
| TCGA_TCGA-HZ-8636 | A |
| TCGA_TCGA-FB-AAPY | A |
| TCGA_TCGA-FB-A4P5 | A |
| TCGA_TCGA-2L-AAQA | A |
| TCGA_TCGA-H8-A6C1 | A |
| TCGA_TCGA-2J-AABP | B |
| TCGA_TCGA-US-A77G | A |
| TCGA_TCGA-IB-A7LX | A |
| TCGA_TCGA-IB-7891 | B |
| TCGA_TCGA-YB-A89D | A |
| TCGA_TCGA-IB-A5SQ | A |
| TCGA_TCGA-FB-AAPQ | A |
| GSE28735geoMatrix_GSM711904 | A |
| GSE28735geoMatrix_GSM711906 | A |
| GSE28735geoMatrix_GSM711908 | A |
| GSE28735geoMatrix_GSM711910 | B |
| GSE28735geoMatrix_GSM711912 | A |
| GSE28735geoMatrix_GSM711914 | A |
| GSE28735geoMatrix_GSM711916 | A |
| GSE28735geoMatrix_GSM711918 | A |
| GSE28735geoMatrix_GSM711920 | A |
| GSE28735geoMatrix_GSM711922 | A |
| GSE28735geoMatrix_GSM711924 | A |
| GSE28735geoMatrix_GSM711926 | B |
| GSE28735geoMatrix_GSM711928 | A |
| GSE28735geoMatrix_GSM711930 | A |
| GSE28735geoMatrix_GSM711932 | A |
| GSE28735geoMatrix_GSM711934 | A |
| GSE28735geoMatrix_GSM711936 | B |
| GSE28735geoMatrix_GSM711938 | B |
| GSE28735geoMatrix_GSM711940 | A |
| GSE28735geoMatrix_GSM711942 | A |
| GSE28735geoMatrix_GSM711944 | B |
| GSE28735geoMatrix_GSM711946 | B |
| GSE28735geoMatrix_GSM711948 | B |
| GSE28735geoMatrix_GSM711950 | B |
| GSE28735geoMatrix_GSM711952 | A |
| GSE28735geoMatrix_GSM711954 | A |
| GSE28735geoMatrix_GSM711956 | A |
| GSE28735geoMatrix_GSM711958 | A |
| GSE28735geoMatrix_GSM711960 | A |
| GSE28735geoMatrix_GSM711962 | A |
| GSE28735geoMatrix_GSM711964 | A |
| GSE28735geoMatrix_GSM711966 | A |
| GSE28735geoMatrix_GSM711968 | A |
| GSE28735geoMatrix_GSM711970 | A |
| GSE28735geoMatrix_GSM711972 | A |
| GSE28735geoMatrix_GSM711974 | A |
| GSE28735geoMatrix_GSM711976 | B |
| GSE28735geoMatrix_GSM711978 | B |
| GSE28735geoMatrix_GSM711980 | A |
| GSE28735geoMatrix_GSM711982 | A |
| GSE28735geoMatrix_GSM711984 | B |
| GSE28735geoMatrix_GSM711986 | A |
| GSE28735geoMatrix_GSM711988 | A |
| GSE28735geoMatrix_GSM711990 | B |
| GSE28735geoMatrix_GSM711992 | A |
| GSE57495geoMatrix_GSM1383778 | A |
| GSE57495geoMatrix_GSM1383779 | B |
| GSE57495geoMatrix_GSM1383780 | A |
| GSE57495geoMatrix_GSM1383781 | A |
| GSE57495geoMatrix_GSM1383782 | A |
| GSE57495geoMatrix_GSM1383783 | A |
| GSE57495geoMatrix_GSM1383784 | A |
| GSE57495geoMatrix_GSM1383785 | A |
| GSE57495geoMatrix_GSM1383786 | A |
| GSE57495geoMatrix_GSM1383787 | B |
| GSE57495geoMatrix_GSM1383788 | B |
| GSE57495geoMatrix_GSM1383789 | A |
| GSE57495geoMatrix_GSM1383790 | A |
| GSE57495geoMatrix_GSM1383791 | B |
| GSE57495geoMatrix_GSM1383792 | A |
| GSE57495geoMatrix_GSM1383793 | B |
| GSE57495geoMatrix_GSM1383794 | B |
| GSE57495geoMatrix_GSM1383795 | A |
| GSE57495geoMatrix_GSM1383796 | B |
| GSE57495geoMatrix_GSM1383797 | A |
| GSE57495geoMatrix_GSM1383798 | A |
| GSE57495geoMatrix_GSM1383799 | B |
| GSE57495geoMatrix_GSM1383800 | A |
| GSE57495geoMatrix_GSM1383801 | A |
| GSE57495geoMatrix_GSM1383802 | A |
| GSE57495geoMatrix_GSM1383803 | A |
| GSE57495geoMatrix_GSM1383804 | A |
| GSE57495geoMatrix_GSM1383805 | A |
| GSE57495geoMatrix_GSM1383806 | A |
| GSE57495geoMatrix_GSM1383807 | A |
| GSE57495geoMatrix_GSM1383808 | A |
| GSE57495geoMatrix_GSM1383809 | A |
| GSE57495geoMatrix_GSM1383810 | A |
| GSE57495geoMatrix_GSM1383811 | B |
| GSE57495geoMatrix_GSM1383812 | B |
| GSE57495geoMatrix_GSM1383813 | A |
| GSE57495geoMatrix_GSM1383814 | A |
| GSE57495geoMatrix_GSM1383815 | A |
| GSE57495geoMatrix_GSM1383816 | B |
| GSE57495geoMatrix_GSM1383817 | A |
| GSE57495geoMatrix_GSM1383818 | A |
| GSE57495geoMatrix_GSM1383819 | B |
| GSE57495geoMatrix_GSM1383820 | A |
| GSE57495geoMatrix_GSM1383821 | B |
| GSE57495geoMatrix_GSM1383822 | A |
| GSE57495geoMatrix_GSM1383823 | A |
| GSE57495geoMatrix_GSM1383824 | A |
| GSE57495geoMatrix_GSM1383825 | B |
| GSE57495geoMatrix_GSM1383826 | A |
| GSE57495geoMatrix_GSM1383827 | A |
| GSE57495geoMatrix_GSM1383828 | A |
| GSE57495geoMatrix_GSM1383829 | A |
| GSE57495geoMatrix_GSM1383830 | A |
| GSE57495geoMatrix_GSM1383831 | A |
| GSE57495geoMatrix_GSM1383832 | A |
| GSE57495geoMatrix_GSM1383833 | B |
| GSE57495geoMatrix_GSM1383834 | B |
| GSE57495geoMatrix_GSM1383835 | B |
| GSE57495geoMatrix_GSM1383836 | B |
| GSE57495geoMatrix_GSM1383837 | A |
| GSE57495geoMatrix_GSM1383838 | B |
| GSE57495geoMatrix_GSM1383839 | A |
| GSE57495geoMatrix_GSM1383840 | A |
| GSE62452geoMatrix_GSM1527105 | A |
| GSE62452geoMatrix_GSM1527107 | A |
| GSE62452geoMatrix_GSM1527109 | A |
| GSE62452geoMatrix_GSM1527111 | B |
| GSE62452geoMatrix_GSM1527113 | A |
| GSE62452geoMatrix_GSM1527115 | A |
| GSE62452geoMatrix_GSM1527117 | A |
| GSE62452geoMatrix_GSM1527119 | A |
| GSE62452geoMatrix_GSM1527121 | A |
| GSE62452geoMatrix_GSM1527123 | A |
| GSE62452geoMatrix_GSM1527125 | A |
| GSE62452geoMatrix_GSM1527127 | B |
| GSE62452geoMatrix_GSM1527129 | A |
| GSE62452geoMatrix_GSM1527131 | A |
| GSE62452geoMatrix_GSM1527133 | A |
| GSE62452geoMatrix_GSM1527135 | A |
| GSE62452geoMatrix_GSM1527137 | B |
| GSE62452geoMatrix_GSM1527139 | B |
| GSE62452geoMatrix_GSM1527141 | A |
| GSE62452geoMatrix_GSM1527143 | A |
| GSE62452geoMatrix_GSM1527145 | B |
| GSE62452geoMatrix_GSM1527147 | B |
| GSE62452geoMatrix_GSM1527149 | B |
| GSE62452geoMatrix_GSM1527151 | B |
| GSE62452geoMatrix_GSM1527153 | A |
| GSE62452geoMatrix_GSM1527155 | A |
| GSE62452geoMatrix_GSM1527157 | A |
| GSE62452geoMatrix_GSM1527159 | A |
| GSE62452geoMatrix_GSM1527161 | A |
| GSE62452geoMatrix_GSM1527163 | A |
| GSE62452geoMatrix_GSM1527165 | A |
| GSE62452geoMatrix_GSM1527167 | A |
| GSE62452geoMatrix_GSM1527169 | A |
| GSE62452geoMatrix_GSM1527171 | A |
| GSE62452geoMatrix_GSM1527173 | A |
| GSE62452geoMatrix_GSM1527175 | A |
| GSE62452geoMatrix_GSM1527177 | B |
| GSE62452geoMatrix_GSM1527179 | B |
| GSE62452geoMatrix_GSM1527181 | A |
| GSE62452geoMatrix_GSM1527183 | A |
| GSE62452geoMatrix_GSM1527185 | B |
| GSE62452geoMatrix_GSM1527187 | A |
| GSE62452geoMatrix_GSM1527189 | A |
| GSE62452geoMatrix_GSM1527191 | B |
| GSE62452geoMatrix_GSM1527193 | A |
| GSE62452geoMatrix_GSM1527196 | A |
| GSE62452geoMatrix_GSM1527198 | A |
| GSE62452geoMatrix_GSM1527199 | A |
| GSE62452geoMatrix_GSM1527200 | B |
| GSE62452geoMatrix_GSM1527202 | A |
| GSE62452geoMatrix_GSM1527204 | A |
| GSE62452geoMatrix_GSM1527205 | B |
| GSE62452geoMatrix_GSM1527207 | A |
| GSE62452geoMatrix_GSM1527209 | A |
| GSE62452geoMatrix_GSM1527210 | A |
| GSE62452geoMatrix_GSM1527212 | B |
| GSE62452geoMatrix_GSM1527213 | A |
| GSE62452geoMatrix_GSM1527215 | B |
| GSE62452geoMatrix_GSM1527216 | B |
| GSE62452geoMatrix_GSM1527218 | A |
| GSE62452geoMatrix_GSM1527219 | A |
| GSE62452geoMatrix_GSM1527220 | A |
| GSE62452geoMatrix_GSM1527223 | B |
| GSE62452geoMatrix_GSM1527225 | A |
| GSE62452geoMatrix_GSM1527227 | B |
| GSE62452geoMatrix_GSM1527228 | B |
| GSE62452geoMatrix_GSM1527230 | B |
| GSE62452geoMatrix_GSM1527232 | B |
| GSE62452geoMatrix_GSM1527234 | B |
| GSE85916geoMatrix_GSM2287304 | A |
| GSE85916geoMatrix_GSM2287305 | B |
| GSE85916geoMatrix_GSM2287306 | A |
| GSE85916geoMatrix_GSM2287307 | B |
| GSE85916geoMatrix_GSM2287308 | A |
| GSE85916geoMatrix_GSM2287309 | A |
| GSE85916geoMatrix_GSM2287310 | B |
| GSE85916geoMatrix_GSM2287311 | A |
| GSE85916geoMatrix_GSM2287312 | B |
| GSE85916geoMatrix_GSM2287313 | A |
| GSE85916geoMatrix_GSM2287314 | A |
| GSE85916geoMatrix_GSM2287315 | A |
| GSE85916geoMatrix_GSM2287316 | A |
| GSE85916geoMatrix_GSM2287317 | B |
| GSE85916geoMatrix_GSM2287318 | B |
| GSE85916geoMatrix_GSM2287319 | B |
| GSE85916geoMatrix_GSM2287320 | A |
| GSE85916geoMatrix_GSM2287321 | B |
| GSE85916geoMatrix_GSM2287322 | A |
| GSE85916geoMatrix_GSM2287323 | B |
| GSE85916geoMatrix_GSM2287324 | B |
| GSE85916geoMatrix_GSM2287325 | B |
| GSE85916geoMatrix_GSM2287326 | A |
| GSE85916geoMatrix_GSM2287327 | A |
| GSE85916geoMatrix_GSM2287328 | A |
| GSE85916geoMatrix_GSM2287329 | A |
| GSE85916geoMatrix_GSM2287330 | B |
| GSE85916geoMatrix_GSM2287331 | A |
| GSE85916geoMatrix_GSM2287332 | B |
| GSE85916geoMatrix_GSM2287333 | A |
| GSE85916geoMatrix_GSM2287334 | B |
| GSE85916geoMatrix_GSM2287335 | A |
| GSE85916geoMatrix_GSM2287336 | B |
| GSE85916geoMatrix_GSM2287337 | A |
| GSE85916geoMatrix_GSM2287338 | A |
| GSE85916geoMatrix_GSM2287339 | A |
| GSE85916geoMatrix_GSM2287340 | A |
| GSE85916geoMatrix_GSM2287341 | A |
| GSE85916geoMatrix_GSM2287342 | A |
| GSE85916geoMatrix_GSM2287343 | B |
| GSE85916geoMatrix_GSM2287344 | A |
| GSE85916geoMatrix_GSM2287345 | A |
| GSE85916geoMatrix_GSM2287346 | A |
| GSE85916geoMatrix_GSM2287347 | A |
| GSE85916geoMatrix_GSM2287348 | B |
| GSE85916geoMatrix_GSM2287349 | A |
| GSE85916geoMatrix_GSM2287350 | B |
| GSE85916geoMatrix_GSM2287351 | A |
| GSE85916geoMatrix_GSM2287352 | A |
| GSE85916geoMatrix_GSM2287353 | B |
| GSE85916geoMatrix_GSM2287354 | B |
| GSE85916geoMatrix_GSM2287355 | A |
| GSE85916geoMatrix_GSM2287356 | A |
| GSE85916geoMatrix_GSM2287357 | A |
| GSE85916geoMatrix_GSM2287358 | A |
| GSE85916geoMatrix_GSM2287359 | B |
| GSE85916geoMatrix_GSM2287360 | A |
| GSE85916geoMatrix_GSM2287361 | A |
| GSE85916geoMatrix_GSM2287362 | A |
| GSE85916geoMatrix_GSM2287363 | A |
| GSE85916geoMatrix_GSM2287364 | A |
| GSE85916geoMatrix_GSM2287365 | A |
| GSE85916geoMatrix_GSM2287366 | A |
| GSE85916geoMatrix_GSM2287367 | A |
| GSE85916geoMatrix_GSM2287368 | B |
| GSE85916geoMatrix_GSM2287369 | A |
| GSE85916geoMatrix_GSM2287370 | B |
| GSE85916geoMatrix_GSM2287371 | A |
| GSE85916geoMatrix_GSM2287372 | A |
| GSE85916geoMatrix_GSM2287373 | A |
| GSE85916geoMatrix_GSM2287374 | B |
| GSE85916geoMatrix_GSM2287375 | A |
| GSE85916geoMatrix_GSM2287376 | A |
| GSE85916geoMatrix_GSM2287377 | A |
| GSE85916geoMatrix_GSM2287378 | A |
| GSE85916geoMatrix_GSM2287379 | A |
| GSE85916geoMatrix_GSM2287380 | A |
| GSE85916geoMatrix_GSM2287381 | A |
| GSE85916geoMatrix_GSM2287382 | A |
| GSE85916geoMatrix_GSM2287383 | A |
